# Discovery of immunotherapy targets for pediatric solid and brain tumors by exon-level expression

**DOI:** 10.21203/rs.3.rs-3821632/v1

**Published:** 2024-01-05

**Authors:** Timothy I Shaw, Jessica Wagner, Liqing Tian, Elizabeth Wickman, Suresh Poudel, Jian Wang, Robin Paul, Selene C. Koo, Meifen Lu, Heather Sheppard, Yiping Fan, Francis O’Neil, Ching C. Lau, Xin Zhou, Jinghui Zhang, Stephen Gottschalk

**Affiliations:** 1 Department of Computational Biology, St. Jude Children’s Research Hospital, Memphis, TN 38105, USA; 2 Department of Bone Marrow Transplantation and Cellular Therapy, St. Jude Children’s Research Hospital, Memphis, TN 38105, USA; 3 Graduate School of Biomedical Sciences, St. Jude Children’s Research Hospital, Memphis, TN 38105, USA; 4 Center for Proteomics and Metabolomics, St. Jude Children’s Research Hospital, Memphis, TN 38105, USA; 5 Department of Pathology, St. Jude Children’s Research Hospital, Memphis, TN 38105, USA; 6 Center for Applied Bioinformatics, St. Jude Children’s Research Hospital, Memphis, TN 38105, USA; 7 The Jackson Laboratory Cancer Center, Farmington, CT, USA.

**Keywords:** pediatric cancer, solid tumors, brain tumors, exon, splice-variants, immunotherapy, CAR T cells

## Abstract

Immunotherapy with CAR T cells for pediatric solid and brain tumors is constrained by available targetable antigens. Cancer-specific exons (CSE) present a promising reservoir of targets; however, these have not been explored and validated systematically in a pan-cancer fashion. To identify CSE targets, we analyzed 1,532 RNA-seq datasets from 16 types of pediatric solid and brain tumors for comparison with normal tissues using a newly developed workflow. We found 2,933 exons in 157 genes encoding proteins of the surfaceome or matrisome with high cancer specificity either at the gene (n=148) or the alternatively spliced (AS) isoform (n=9) level. Expression of selected AS targets, including the EDB domain of FN1 (EDB), and gene targets, such as COL11A1, were validated in pediatric PDX tumors. We generated CAR T cells specific to EDB or COL11A1 and demonstrated that COL11A1-CAR T-cells have potent antitumor activity. The full target list, explorable via an interactive web portal (https://cseminer.stjude.org/), provides a rich resource for developing immunotherapy of pediatric solid and brain tumors using gene or AS targets with high expression specificity in cancer.

## INTRODUCTION

Immunotherapy with T cells expressing chimeric antigen receptors (CARs) holds the promise to improve outcomes for pediatric solid tumors, including brain tumors^1^. However, in contrast to CAR T-cell therapy for CD19-positive hematological malignancies, the antitumor activity of CAR T cells for solid and brain tumors has been limited^2^. Lack of efficacy is most likely multifactorial, including limited T-cell fitness, inefficient homing to tumor sites, the hostile tumor microenvironment, and a limited array of targetable antigens^2,3^.

The majority of approaches for identifying new surfaceome targets for pediatric solid tumors have largely relied on differential gene expression analysis^4^, often in a specific cancer type^5–7^. These approaches may lead to missed opportunities for finding pan-cancer targets that are effective across multiple types, some of which may arise from alternative splicing. We are interested in identifying cancer-specific exons (CSEs) herein referring to those with high expression in tumor but restricted and limited expression in normal tissues; some of which encode tumor-associated antigens which can serve as CAR targets.^8^ To discover CSE targets for immunotherapy for pediatric solid and brain tumors, we analyzed 1,532 RNA-seq samples from the St. Jude/Washington University Pediatric Cancer Genome Project (PCGP), the National Cancer Institute (NCI) Therapeutically Applicable Research to Generate Effective Treatment (TARGET), and St. Jude Children’s Research Hospital (St. Jude) Cloud^9^ real-time clinical genomics data (ClinGen). We identified a total of 2,933 cancer-specific exons in 157 genes encoding surfaceome or matrisome, including 9 alternatively spliced (AS) isoform targets. Using pediatric cancer cell lines and patient-derived xenograft mouse models (PDXs), we experimentally validated the expression of the surface antigen of procollagen 11A1 (COL11A1) and two extracellular matrix (ECM) proteins, VCAN isoform 1 and fibronectin (FN1) in the EDB domain. All three targets were present in a broad spectrum of pediatric solid tumors and brain tumor types. Finally, we show that CAR T cells targeting COL11A1 or EDB demonstrate potent antitumor activity against pediatric sarcoma.

## RESULTS

### Discovery of CSE in pediatric solid and brain tumors

**Figure 1:**
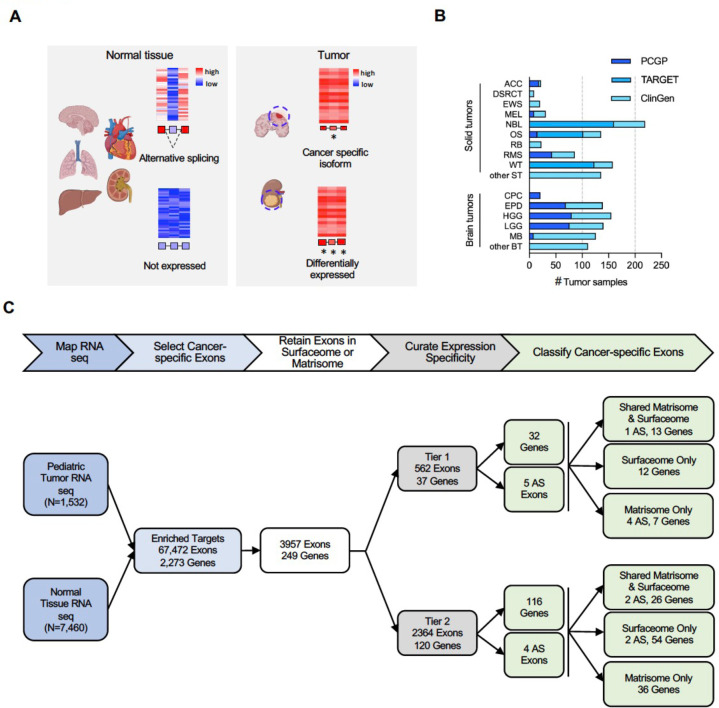
Discovery of CSEs as immunotherapy targets in pediatric solid and brain tumors. (**A**) Schematic illustration of the concept of exploiting CSEs, which include both gene-level and alternatively spliced exons as targets for immunotherapy (created with BioRender software). (**B**) Pediatric solid and brain tumor RNA-seq data sets used for discovery. The sample count in each tumor type is colored by the data source (i.e., PCGP, TARGET, and St. Jude’s ClinGen). Nine major types of solid tumors are marked by their abbreviations as follows: adrenocortical carcinoma (ACC), desmoplastic round cell tumor (DSRCT), Ewing sarcoma (EWS), melanoma (MEL), neuroblastoma (NBL), osteosarcoma (OS), retinoblastoma (RB), rhabdomyosarcoma (RMS), and Wilms tumor (WT). Rare solid tumors are binned into the category of other solid tumors (other ST). Five brain tumor types are shown by their abbreviation as follows: choroid plexus carcinoma (CPC), ependymoma (EPN), high-grade glioma (HGG), low-grade glioma (LGG), medulloblastoma (MB). Rare brain tumors are binned into other brain tumors (other BT). (**C**) Analysis workflow (top) and resulting data (bottom) involving the following steps: 1) Quantify exon-level expression by RNA-seq mapping; 2) Select exons highly expressed in tumor but not normal tissues; 3) Retain exons from surfaceome/matrisome; 4) Perform curation expression specificity to remove artifacts and to categorize Tier 1 and Tier 2 candidates representing those without and with expression in adjacent/critical tissues (e.g., brain, liver, bone marrow); Tier 1 targets also require to have low proteomics expression in GTEx. 5) Classify targets into AS exons versus gene-level.

To identify CSEs (Fig 1A), we analyzed the cancer-specific transcription profiles of RNA-seq data from 840 solid and 692 brain tumor samples (Fig 1B). Major types of solid tumors included i) adrenocortical carcinoma (ACC, n=22), ii) desmoplastic round cell tumor (DSRCT, n=9), iii) Ewing sarcoma (EWS, n=20), iv) melanoma (MEL, n=31), v) neuroblastoma (NBL, n=219), vi) osteosarcoma (OS, n=136), vii) retinoblastoma (RB, n=23), viii) rhabdomyosarcoma (RMS, n=86), ix) Wilms tumor (WT, n=158), x) other solid tumors (other ST, n=136). Major types of brain tumor include i) choroid plexus carcinoma (CPC, n=21), ii) ependymoma (EPN, n=139), iii) high grade glioma (n=155), iv) low grade glioma (LGG, n=140), v) medulloblastoma (MB, n=126), and vi) other brain tumors (other BT, n=111). For normal tissue comparison, we analyzed 7,460 RNA-seq samples across 30 normal tissues from the Genotype-Tissue Expression (GTEx) database (**Supplementary Fig 1**).

CSEs were identified by an analytical pipeline involving the following five main steps (Fig 1C): 1) map RNA-seq data of 1,532 tumor samples and 7,460 normal tissue samples, 2) select cancer-specific exons based on enriched expression in tumors, 3) retain exons that are present in proteins present on the cell surface (surfaceome) or extracellular matrix (ECM; matrisome)^10–12^, 4) curate targets based on expression specificity in cancer, and 5) classify CSEs according to aberrant gene-level transcription or AS isoforms in tumors.

**Extended Data Figure 1.**
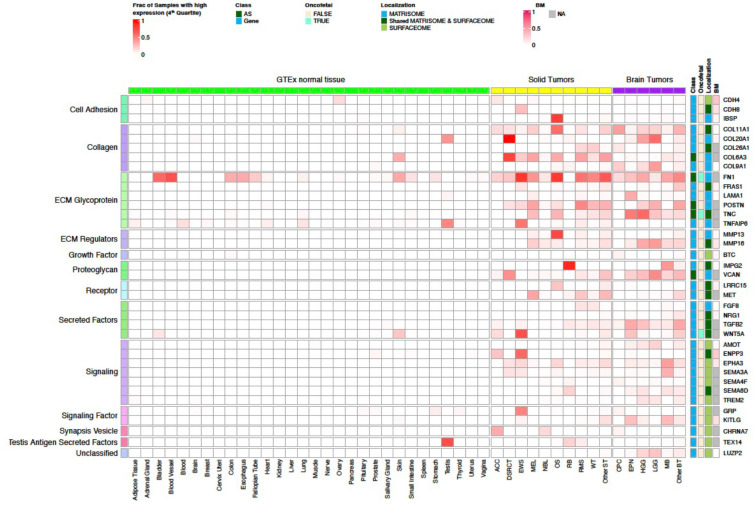
Prevalence of samples with high expression of Tier 1 targets. Heatmap has the same layout as Figure 2; with the color scale represents the proportion of samples shown high expression (4^th^ quartile) in a tumor type or in a normal tissue type.

**Extended Data Figure 2.**
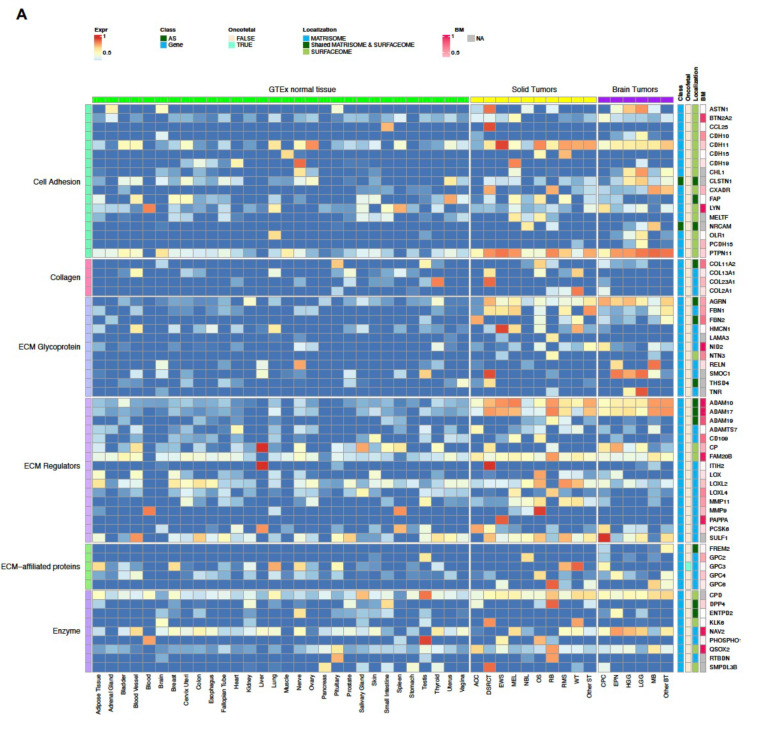

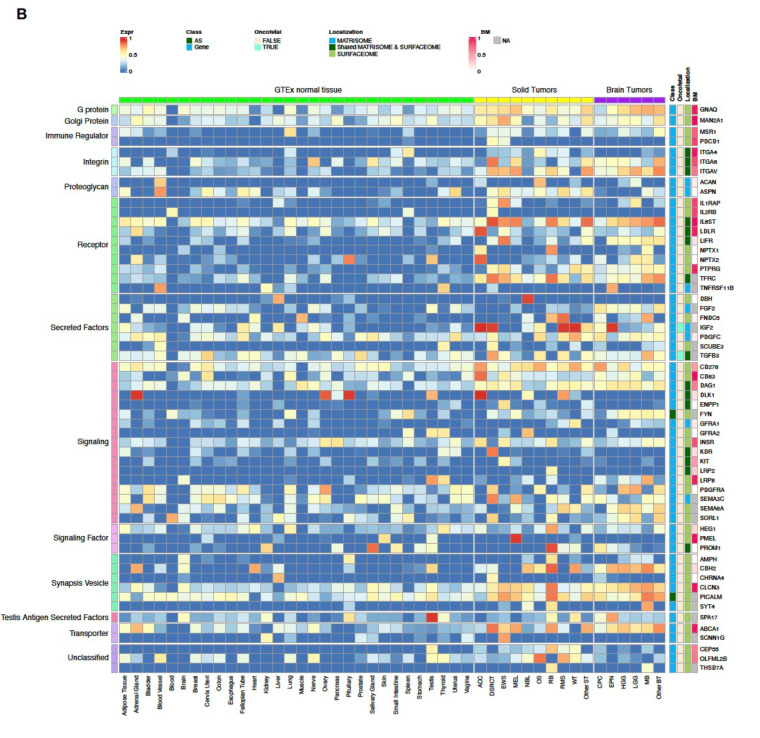
Normalized expression of Tier 2 targets across pediatric cancer types and normal tissues. The heatmap spilt into panels A and B has the same layout as Figure 2.

**Extended Data Figure 3.**
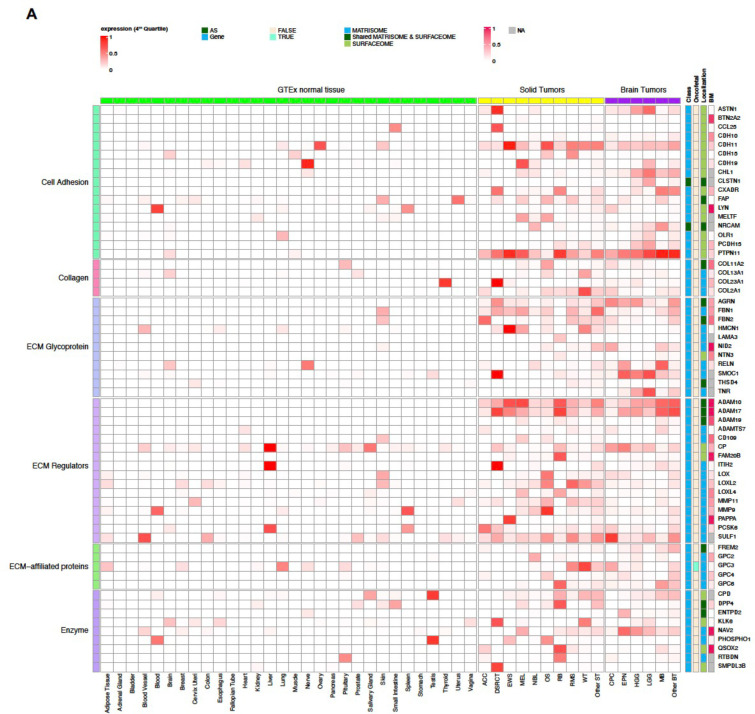

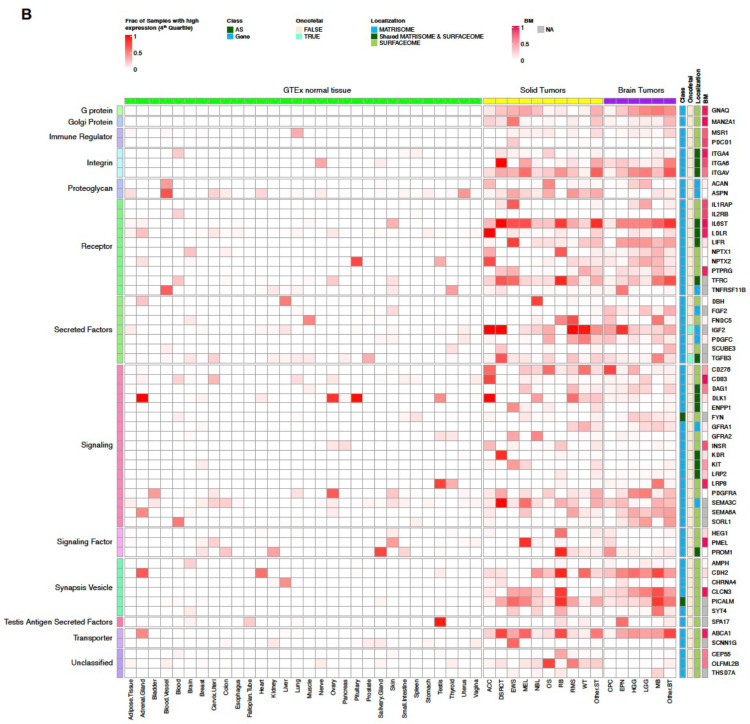
Prevalence of samples with high expression of Tier 2 targets. Heatmap spilt into panels A and B has the same layout as Figure 2; with the color scale represents the proportion of samples shown high expression (4^th^ quartile) in a tumor type or in a normal tissue type.

**Figure 2.**
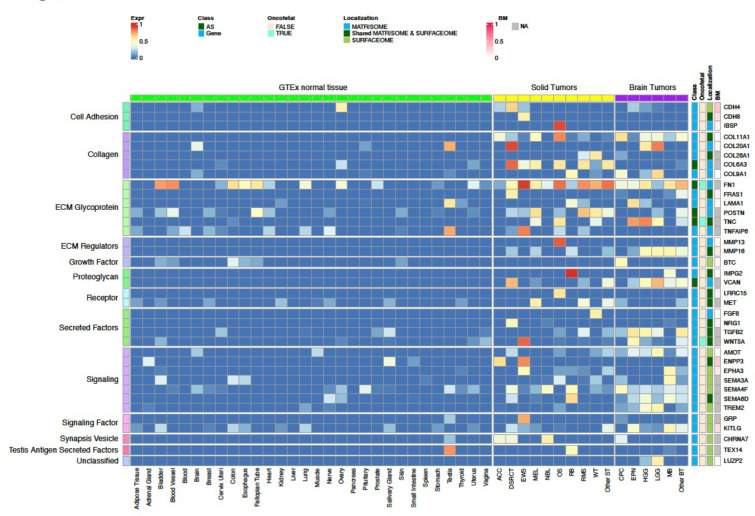
Normalized expression of Tier 1 targets across pediatric cancer types and normal tissues. The heatmap uses a blue-red color scale to display the mean expression rank (range 0–1) of exon FPKM value of RNA-seq samples profiled for a normal tissue or a specific tumor type. 37 Tier 1 targets are grouped by their biological functions on the left, while the gene names, status on AS exon, oncofetal protein, cellular localization, and expression prevalence in normal bone marrow are shown on the right. One representative exon is shown for a gene-level target.

Our CSE pipeline resulted in the identification of 67,472 exons in 2,273 genes, which were enriched in tumors compared to normal tissues. Of these, 3,964 exons in 249 genes belonged to the surfaceome or matrisome. We further classified these into Tier 1 and Tier 2 targets (Figs 2; Extended Data Figs 1–3**; Supplementary Table 1**) with Tier 1 targets having minimal expression in matching normal tissue types and vital organs such as brain, liver, and lung. Gene-level Tier 1 targets were additionally required to have low expression in normal bone marrow samples using a logistic regression model that we previously developed^13^. To ensure Tier 1 targets having high and low protein abundance in tumor and normal tissues, respectively, we further analyzed PDX and GTEx proteomics data resulting in 37 Tier 1 and 120 Tier 2 targets.

We identified 16 AS in 9 genes (7 genes with 1 AS, 1 gene with 2 AS, and 1 gene with 7 AS) (**Supplementary Table 1**). For the two genes in which we identified >1 AS, we selected the most differential AS for the final CSE list, which included 9 AS (5 [Tier 1]; 4 [Tier 2]) and 148 gene-level targets (Fig 1C). Forty-two CSEs were present in the matrisome and surfaceome (AS: 1 [Tier 1], 2 [Tier 2]; gene-level: 13 [Tier 1], 26 [Tier 2]), 68 only in the surfacesome (AS: 2 [Tier 2]; gene-level: 12 [Tier 1], 54 [Tier 2]), and 47 only in the matrisome (AS: 4 [Tier 1]; gene-level: 7 [Tier 1], 36 [Tier 2]). Protein expression of Tier 1 and 2 targets was confirmed using published proteomic data sets^14,15^. To assess whether expression of the 9 AS targets was associated with variants at the splice sites, we analyzed 504 samples with matched tumor WGS data in our pediatric cancer cohort and did not find any significant association in two genes (FN1, VCAN) that harbored such variants (**Supplementary Table 2**). No such variants were found in the other seven genes. This indicates that the AS targets were caused by transcription deregulation instead of genomic variants in pediatric cancer samples that we analyzed.

### Landscape of CSE immunotherapeutic targets in pediatric cancers

Tier 1 and 2 targets identified by our analysis encoded proteins with diverse biological functions such as cell adhesion, collagen, ECM, receptor, and signaling factors (Fig 2; Extended Data Figs 1–3). Interestingly, 56.7% (89 out of 157) of the targets were associated with the matrisome, indicating that ECM proteins provide a rich source of tumor-specific antigens. Of the 9 AS targets, three (FN1, TNC, and COL6A3) showed high expression in OS, and all three were confirmed by full-length transcriptome sequencing of 3 OS patient samples (**Supplementary Fig 2**).

To our knowledge, amongst the 157 targets, 11 (CD83, CD276 (B7-H3), FAP, FN1, GPC2, GPC3, IL1RAP, KDR, KIT, MET, PROM1 (CD133)) have been explored as CAR targets in preclinical studies^5,8,16–26^, while the remaining 146 (93%) are novel. Of the known targets, 5 (FAP, B7-H3, GPC3, KDR, CD133) have been or are actively being explored in clinical studies. Six known oncofetal proteins were also on the target list, which includes AS isoforms of FN1 and TNC as well as gene-level targets TGFB2, WNT5A, GPC3 and IGF2. Given their limited expression in normal tissue beyond the fetal development stage, these oncofetal proteins should be considered high priority targets. Similarly, testis-restricted targets such as SPA17, TEX14, LAMA1, SMOC1, TNFAIP6, GPC2, and COL20A1 (Fig 2; Extended Data Fig 2) may also be leveraged for their cancer-specificity. Indeed, GPC2-CAR T cells have already been developed for NB^5,27^.

Approximately 30% (49 of 157) of the targets are highly expressed in both solid and brain tumors at high prevalence (≥25%) in at least one tumor type (Extended Data Figs 1, 3), highlighting the potential for developing pan-cancer targets. These include 6 of the 9 AS targets (FN1, TNC, NRCAM, PICALM, FYN, VCAN). Specifically, FN1 encodes fibronectin which is involved in cell adhesion and migration processes including embryogenesis, wound healing, blood coagulation, host defense, and metastasis^28^. The identified FN1 AS target encodes the alternatively spliced extra domain B of FN1 (EDB)^29^ which is highly expressed in all solid and brain tumor types except RB, with the highest prevalence in OS, EWS, RMS, WT, MEL, HGG and EPN (Extended Data Fig 1). TNC encodes an extracellular matrix protein that plays a role during normal development, including neural migration, as well as tumorigenesis^30,31^. The TNC AS target encodes the alternatively spliced C domain of TNC^32^ with high expression detected at high prevalence in HGG, EPN, OS and MEL (Extended Data Fig 1). VCAN is a member of the aggrecan/versican proteoglycan family and is involved in cell adhesion, proliferation, proliferation, migration and angiogenesis. Mutations in VCAN Mutations can cause Wagner syndrome type 1^33^. The VCAN AS target encodes VCAN isoform 1 (VCAN1), which has the highest expression levels in LGG, HGG, and DSRCT (Extended Data Fig 1). Pan-cancer gene-level targets include COL11A1, which encodes one of the two alpha chains of type XI collagen and is expressed at high prevalence in OS and CPC (Extended Data Fig 1). Mutations in COL11A1 are associated with type II Stickler syndrome and with Marshall syndrome^34^. GPC3 which is highly expressed in RMS, WT, and CPC (Extended Data Fig 3A,B), is a member of glypican family and regulates the signaling of WNTs, Hedgehogs, fibroblast growth factors, and bone morphogenetic proteins. Loss of function mutations in GPC3 can cause Simpson-Golabi-Behmel syndrome^35^. CD276 which is highly expressed in ACC, OS, WT, and HGG (Extended Data Fig 3A,B) belongs to the immunoglobulin superfamily and regulates T-cell-mediated immune responses^36,37^.

### Validation of cell surface expression of selected CSEs in patient-derived xenograft (PDX) models

**Extended Data Figure 4.**
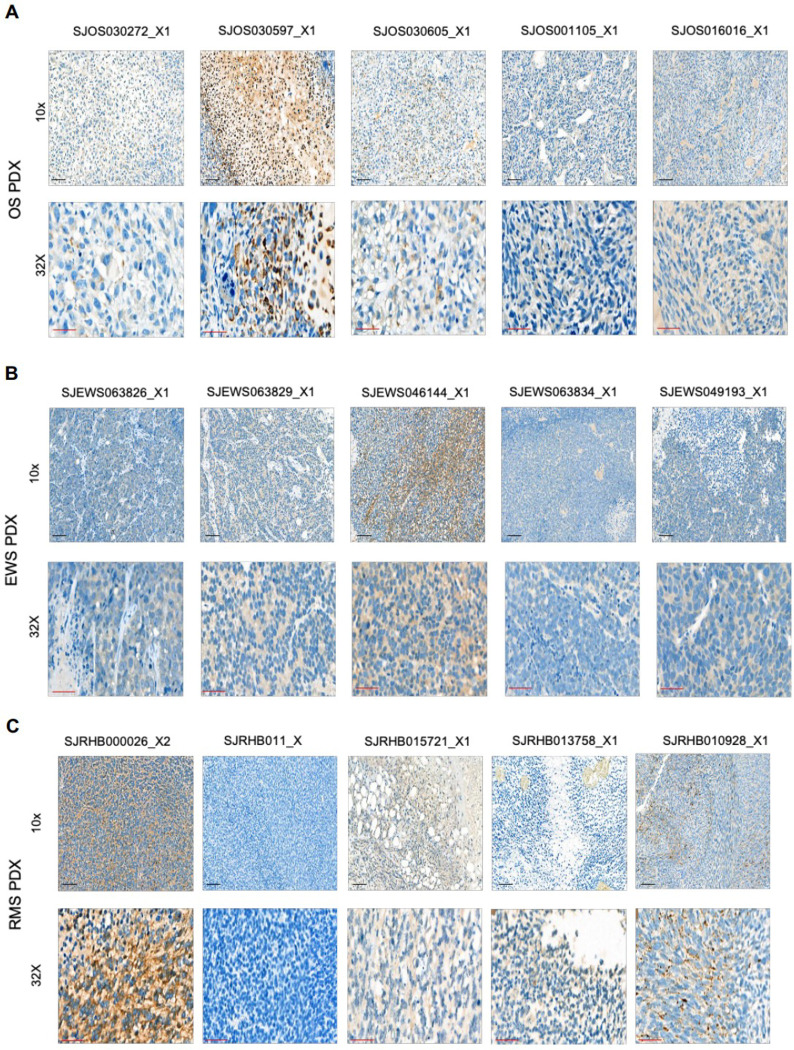
Representative IHC images of COL11A1 expression in OS, EWS, and RMS PDX samples. The COL11A1-specific mAb 1e8.33 was used to detect COL11A1 by IHC. (**A**) OS, (**B**) EWS, (**C**) RMS. For each panel: top row: 10x magnification; scale bar, 100 μm (black). Bottom row: 32x magnification; scale bar, 50 μm (red).

**Extended Data Figure 5.**
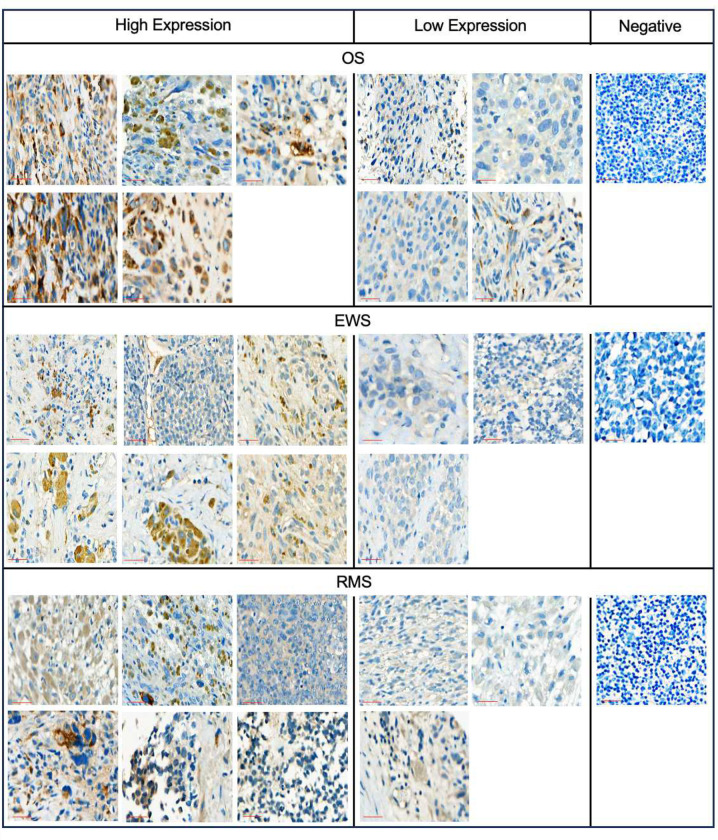
Representative IHC images of COL11A1 expression in primary OS, EWS, and RMS samples. The COL11A1-specific mAb 1e8.33 was used to detect COL11A1 by IHC; 18 OS, 11 EWS, and 37 RMS were analyzed. Ten representative, individual tumors are show for OS, EWS, and RMS. 32x magnification; scale bar, 50 μm (red).

**Extended Data Figure 6.**
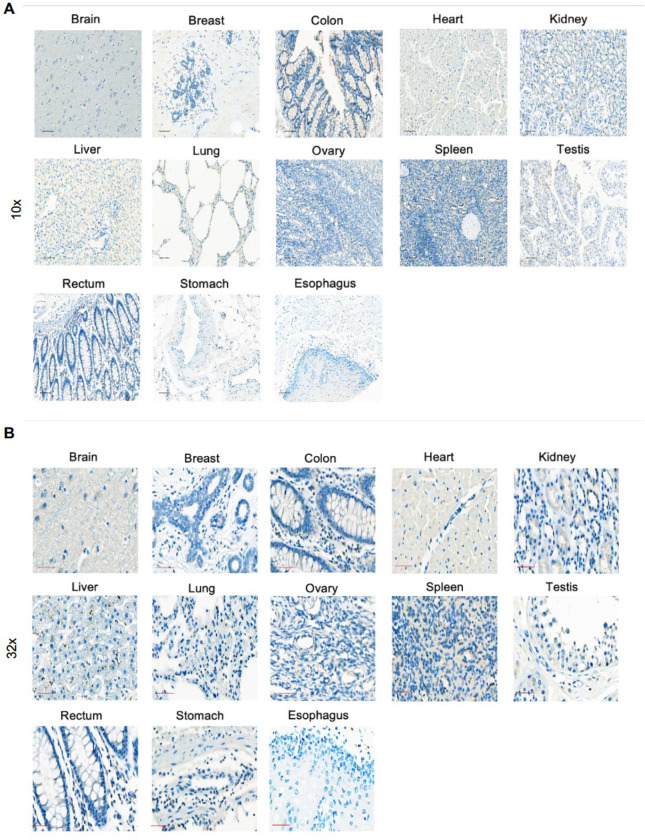
Representative IHC images of COL11A1 expression in normal healthy tissues samples. The COL11A1-specific mAb 1e8.33 was used to detect COL11A1 by IHC. (**A**) Images, 10x magnification; scale bar, 100 μm (black). (**B**) Images, 32x magnification; scale bar, 50 μm (red).

**Figure 3.**
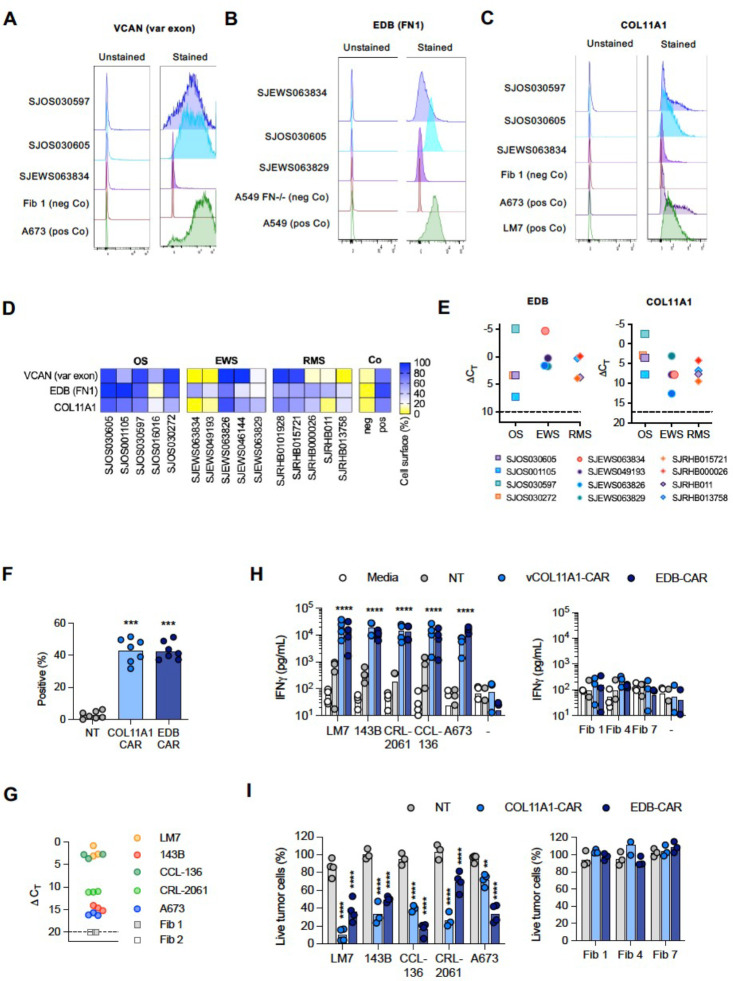
COL11A1-CAR T cells have potent antitumor activity *in vitro*. (**A-C**) Representative histogram of cell surface expression of 3 out of 5 PDX samples for VCAN (variable (var) exon), FN1 (EDB), and COL11A1. Tumor cell lines (A673, A549, LM7) served as positive controls and normal fibroblasts (Fib 1) or A549 FN−/− as negative controls (Co). (**D**) Heat map displaying cell surface expression in PDX samples as determined by flow cytometry. (**E**) RTqPCR for EDB or COL11A1 gene expression performed on PDX samples. Delta CT calculated relative to GAPDH. Dashed line: threshold of positivity based on qPCR results of antigen negative cells. (**F**) CAR expression determined by flow cytometry using an anti-mouse IgG F(ab’)2 (n=7, t-test, ***p<0.001). (**G**) COL11A1 expression of LM7, 143B, CCL-136, CRL-2061, and A673 tumor cells, and two primary fibroblast cell lines (Fib 1, Fib 2) determined by RT-qPCR. Triplicates for each cell line are shown; COL11A1 expression in fibroblast was undetectable and their DCT value was set as 20. (**H**) NT or COL11A1-CAR T-cells were incubated at a 2:1 E:T ratio for 48 hours with tumor cells or primary fibroblasts. Media only samples served as controls. IFNγ in culture media was determined by ELISA (n=3–4 donors, two-way ANOVA of log-transformed data comparing against the NT of each tumor type to Col11A1 of each tumor type; ****p*<*0.0001. All fibroblast experiments were non-significant. (**I**) Cytolytic activity of NT or COL11A1-CAR T-cells at an E:T ratio of 4:1 for 72 hours against GFP.ffluc-expressing tumor cells or primary fibroblasts (n=3–4 donors, two-way ANOVA, comparing against the NT of each tumor type to COL11A1 of each tumor type **p<0.01,****p<0.0001). All fibroblast experiments were non-significant.

Validation was carried out on three Tier 1 targets, FN1, VCAN1, COL11A1, which are expressed in a broad spectrum of pediatric brain and solid tumors based on our analysis. For VCAN1 and EDB, we took advantage of mAbs that recognize the part of the molecule that is encoded by the differentially expressed exon and performed flow cytometric analysis of 15 pediatric PDX samples (5 OS, 5 EWS, 5 RMS; **Supplementary Table 3**). VCAN1, EDB, and COL11A1 were expressed in >50% of tumor cells in 8 or 9/15 PDX samples (Fig 3A–D). In addition, for COL11A1, we performed immunohistochemistry (IHC) on the PDX samples as well as primary tumor samples. For the PDX sample, there was concordance between flow cytometric and IHC analysis with only one flow+/IHC− tumors (Extended Data Fig 4). Of the primary tumors, 12/18 OS, 7/11 EWS, and 14/37 RMS samples highly expressed Col11A1 (Extended Data Fig 5); 5/18 OS, 3/11 EWS, and 10/37 RMS tumor samples showed low expression, respectively. All stained normal tissues remained negative (Extended Data Fig 6). We confirmed EDB and COL11A1 expression in all tumors (100%) by RT-qPCR in 12/12 PDX samples analyzed (4/4 OS, 4/4 EWS, 4/4 RMS) (Fig 3E). Finally, we evaluated COL11A1 expression using publicly available single-cell RNA-seq data generated from 11 tumor samples^38^ and confirmed its presence in 10 out of 11 tumor samples (**Supplementary Fig 3**).

### COL11A1-CAR and EDB-CAR T cells have antitumor activity against multiple types of pediatric sarcoma

**Extended Data Figure 7.**
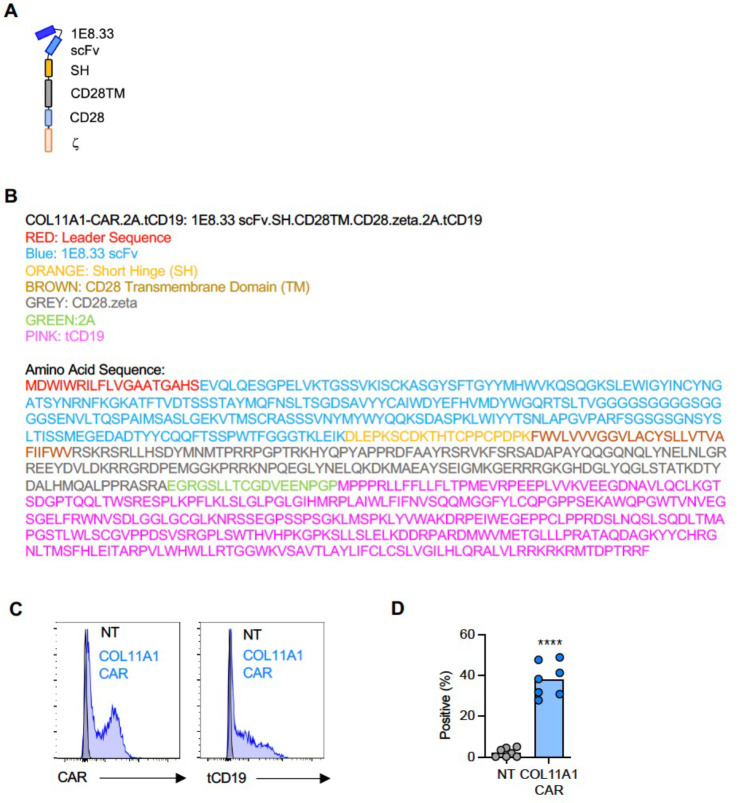
Generation of COL11A1-CAR T cells. (**A**) Structure of COL11A1-CAR: 1E8.33 scFv, M13 short hinge, CD28 transmembrane and costimulatory domain, and a CD3 zeta signaling domain. (**B**) Amino acid sequence of gene encoding COL11A1-CAR-2A-tCD19. (**C**) Representative FACS plots of transduced T cells. Left panel: detection of CAR with anti-mouse IgG (Fab’)2 fragment; Right panel: detection of tCD19 with anti-CD19. (**D**) Summary data for tCD19 expression, n=7, t-test, ****p<0.0001.

We focused on developing a CAR T-cell therapy approach for COL11A1, a novel target identified in this study. In addition, we extended our previous study, in which we had demonstrated that T cells expressing a functional CAR with an EDB-specific single chain variable fragment (scFv) antigen binding domain^39^ (EDB-CAR T cells), recognize and kill one OS (LM7) and one EWS (A673) cell line^19^. We generated a COL11A1.CD28.z-CAR (COL11A1-CAR) with a COL11A1-specific scFv derived from the 1E8.33 mAb, which was raised against a peptide sequence that is unique for COL11A1^40^ (Extended Data Fig 7A,B). COL11A1- and EDB-CAR T cells were generated by retroviral transduction and expression were confirmed by flow cytometry (Fig 3F; Extended Data Fig 7C,D). We performed 48-hour co-culture assays with COL11A1-positive pediatric tumor cell lines (OS: LM7, 143B; RMS: CCL-136, CRL-2061; EWS: A673) and COL11A1-negative primary fibroblasts (Fig 3G).

COL11A1- and EDB-CAR T cells produced significant amounts of IFNγ in comparison to NT T cells only in the presence of antigen-positive tumor cells (Fig 3H). Likewise, both CAR T-cell populations had significant cytolytic activity against antigen-positive tumor cells in comparison to NT T cells in a standard cytotoxicity assay, confirming specificity (Fig 3I). To confirm that the newly generated COL11A1-CAR is antigen-specific, we performed additional orthogonal assays. COL11A1-CAR T cells did not recognize 143B cells in which COL11A1 was knocked out (KO) by CRISPR/Cas9 gene editing, and T cells expressing a non-functional COL11A1-CAR with mutated immunoreceptor tyrosine-based activation motifs (ITAMs) did not kill wildtype 143B cells (**Supplementary Fig 4**).

**Figure 4.**
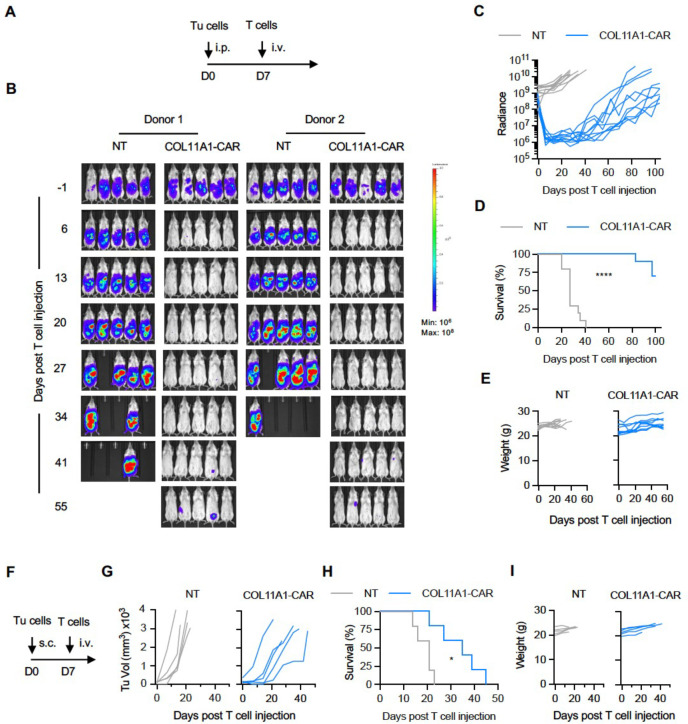
COL11A1-CAR T cells have potent antitumor activity *in vivo*. (**A**) Schematic of LM7 OS animal experiment. Day 0: i.p. injection of 1×10^6^ LM7.GFP.ffluc cells; Day 7: i.v. injection of 3×10^6^ NT or CAR T-cells (n=10 mice per group; 2 different T-cell donors (5 mice per donor). (**B**) Representative bioluminescence images. (**C**) Quantitative bioluminescence data (Radiance: photons/sec/cm^2^/sr). (**D**) Kaplan-Meier survival curve, log-rank (Mantel-Cox) test; ****p<0.0001. (**E**) Weight (g) of mice. (**F**) Schematic of A673 EWS animal experiment. Day 0: s.c. injection of 1×10^6^ A673 cells; Day 7: i.v. injection of 3 × 10^6^ NT or CAR T-cells (n=5 mice per group). (**G**) Tumors measured weekly by caliper measurements. (**H**) Kaplan-Meier survival curve, log-rank (Mantel-Cox) test, *p<0.01. (**I**) Weight (g) of mice.

In the final set of experiments, we evaluated the antitumor activity of COL11A1-CAR T cells *in vivo*. We first utilized our established osteosarcoma model where LM7.GFP.ffLuc cells were injected intraperitoneally (i.p.) into NSG mice followed by one single i.v. dose of 3×10^6^ COL11A1-CAR or NT T cells on day 7 (Fig 4A)^41^. COL11A1-CAR T cells had significant anti-tumor activity as judged by bioluminescence imaging in 10/10 mice in comparison to NT T cells, which had no antitumor activity (Fig 4B,C). This resulted in significant median survival advantage of >100 days post COL11A1-CAR T-cell infusion (Fig 4D), and surviving mice had no clinical evidence of xenogeneic graft versus host disease as judged by inspection of their fur coat and absence of weight loss (Fig 4E). Since tumors eventually recurred, we explored mechanisms of tumor recurrence using the same model with LM7 cells and GFP.ffLuc-expressing CAR or NT T cells (**Supplementary Fig 5A**). We observed CAR T cell expansion but limited persistence, and decreased expression of COL11A1 on day 65 post tumor cell injection (**Supplementary Fig 5B-E**), indicating that tumor recurrence is most likely due to both mechanisms.

We confirmed the antitumor activity of COL11A1-CAR T cells using our subcutaneous EWS (A673) model^41^ in which tumor-bearing mice received one single i.v. dose of 1×10^6^ COL11A1-CAR or NT T cells on day 7 (Fig 4F). COL11A1-CAR T cells had robust antitumor activity, resulting in a significant survival advantage in comparison to NT T cells (Fig 4G–I).

### Exploring CSE targets on the CSE-miner data portal

**Figure 5.**
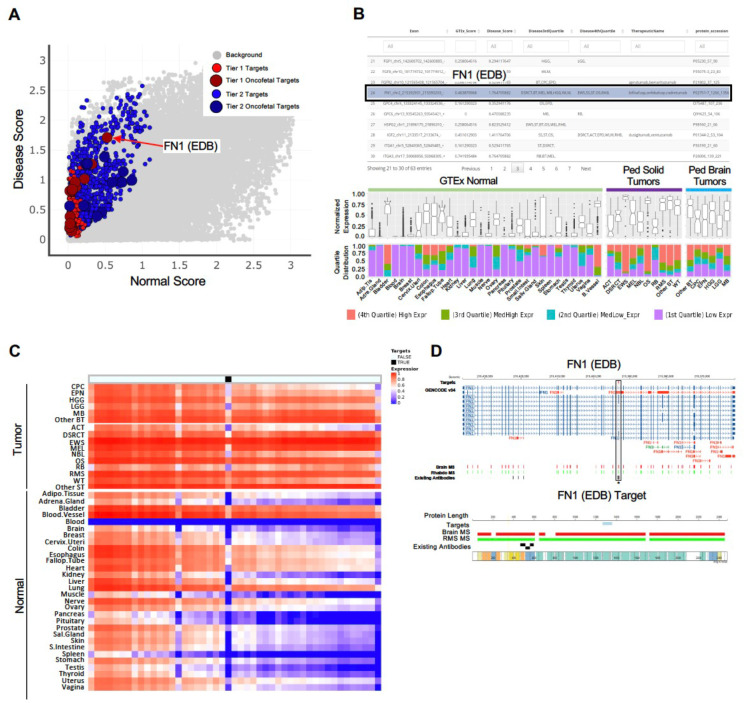
Navigation and visualization with the CSE Miner web portal. An alternatively spliced exon in FN1 encoding the FNB isoform is highlighted to illustrate the visualization features. On the portal (https://cseminer.stjude.org/), users can explore Tier 1 and Tier 2 candidates through the (**A**) pan-target view, (**B**) table view, (**C**) heatmap view, and (**D**) gene view. (**A**) All candidates can be visualized through a two-dimensional scatter plot showing the normalized mean exon expression (details in Methods) across pediatric tumor samples (x-axis) and GTEx normal samples (y-axis). (**B**) On the table view, the distribution of exon expression in normal tissues and tumor types can be examined in percentile (middle panel) and quartiles (bottom panel). (**C**) Heatmap showing exon expression across normal and disease tissue types within a candidate gene. A user can select a transcript of interest (the transcript ENST00000432072.6 was selected for FN1) from the list shown in the left panel. Heatmap represents a mean normalized rank expression for each tissue type (right panel). (**D**) A gene view which can toggle between a genomic view highlighting the exons or a protein view displaying the relevant protein domains.

We developed a web-based data portal, CSE-miner (https://cseminer.stjude.org/) to enable biomedical researchers to explore all targets identified in this study. The data portal includes rich visualization features to allow evaluation of omics data used for CSE identification, along with ancillary information useful for designing future experiments. To illustrate the functionality of the data portal, we used the EDB exon of FN1 as an example. Each target can be explored using four different views as follows: 1) A pan-target scatter plot for prioritizing targets based on the relative expression of tumor samples and normal tissues (Fig 5A); 2) a table view for selecting a CSE of interest to examine its expression pattern across all tumor types and normal tissues (Fig 5B); 3) a heatmap view showing the relative expression in tumor and normal samples across all exons within the gene, highlighting identified CSE targets (Fig 5C); and 4), a gene view which can toggle between a genome view highlighting the specific exons, and a protein view highlighting the domains encoded by the identified targets, along with examples of associated antibody binding regions and proteome expression using mass spectrometry data from CPTAC pediatric brain tumors^14^ and St. Jude’s RMS xenograft tumors^15^ (Fig 5D).

**Extended Data Figure 8.**
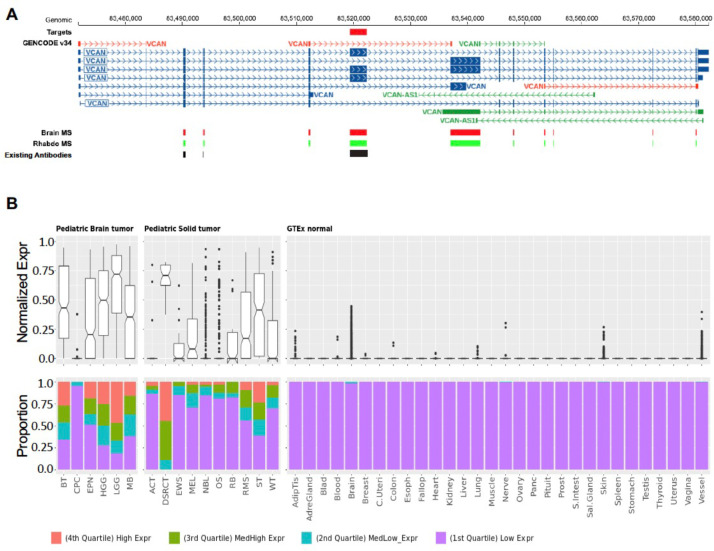
Gene expression pattern of a VCAN AS exon target across tumor types and normal tissues. (**A**) Gene structure of VCAN. The AS exon identified as Tier1 target is shown as a red rectangle. (**B**) Normalized expression shown high expression in tumor and no expression in normal tissues. Boxplot showing the exon expression in rank normalized percentile in the panel above and bar plot showing the quartile distribution across solid and brain tumors in the panel below. Both (**A**) and (**B**) were generated from CSEMiner data portal.

**Extended Data Figure 9.**
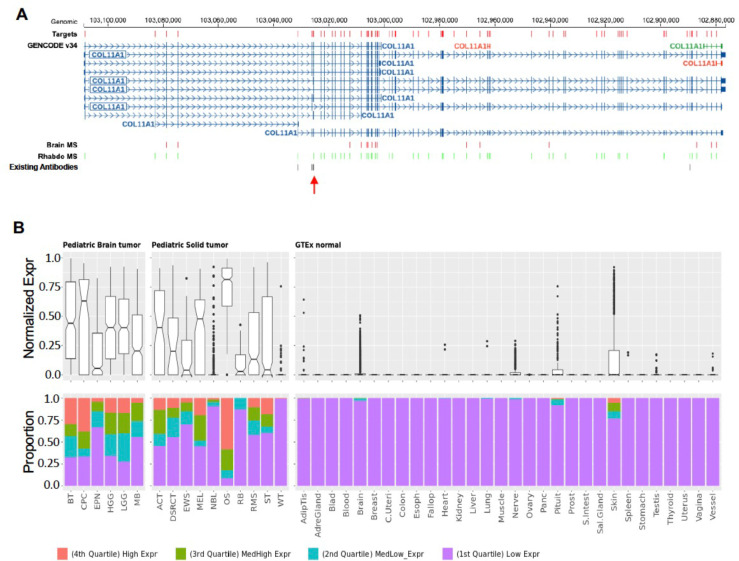
Expression pattern of COL11A1 in tumor types and normal tissues. COL11A1 is a gene-level target and is transcribed in reverse orientation of the reference human genome. (**A**) Gene structure of COL11A1. The exon selected for gene expression display in panel B is marked by a red arrow. (**B**) Normalized expression shown high expression in tumor and no expression in normal tissues. Boxplot showing the exon expression in rank normalized percentile in the panel above and bar plot showing the quartile distribution across solid and brain tumors in the panel below. Both panels were generated from CSEMiner web portal. Both (**A**) and (**B**) were generated from CSEMiner data portal.

**Extended Data Figure 10.**
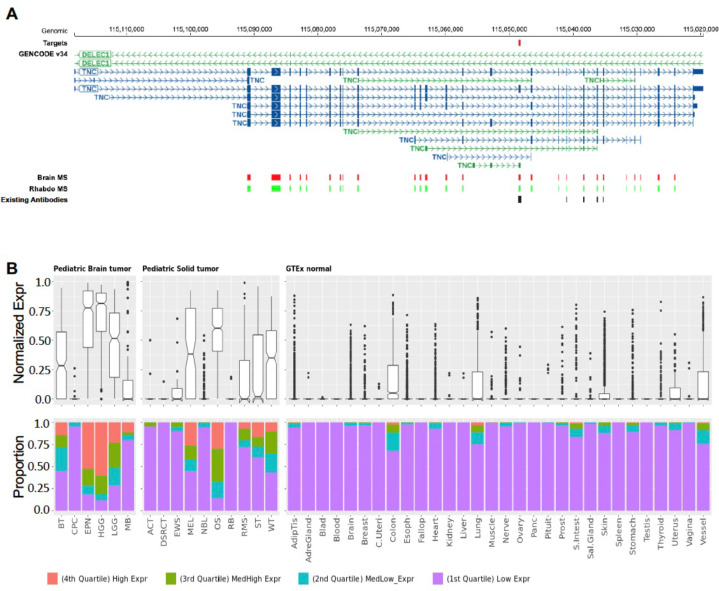
Gene expression pattern of a TNC AS exon target across tumor types and normal tissues. (**A**) Gene structure of TNC that is transcribed in reverse orientation of the reference human genome. The AS exon identified as Tier1 target is shown as a red rectangle. (**B**) Normalized expression shown high expression in tumor and no expression in normal tissues. Boxplot showing the exon expression in rank normalized percentile in the panel above and bar plot showing the quartile distribution across solid and brain tumors in the panel below. Both (**A**) and (**B**) were generated from CSEMiner data portal.

The visualization features implemented in CSEminer were designed to support target prioritization, which requires verifying high-level expression in tumor types and limited expression in normal tissues. This is facilitated by a box plot of normalized expression values and a bar graph of quartile distribution across tumor types and normal tissue types implemented in the table view. Additionally, the gene view enables distinguishing a gene-level target from an AS-exon target, while additional information (e.g., mAb availability) helps with planning future experiments. We illustrated these visualization features for three additional examples that were evaluated for selection of high priority targets: VCAN, COL11A1, and TNC (Extended Data Figs 8–10).

## DISCUSSION

In this study, we describe the first pan-cancer analysis we know of for discovery of CSEs as potential targets for immunotherapy for pediatric solid and brain tumors. Using the large RNA-seq datasets generated by multiple genomic initiatives, we identified 157 gene-level or alternatively spliced exon targets encoding members of surfaceome or matrisome. These targets were further categorized into Tier 1 (n=37) or Tier 2 (n=120), requiring that Tier 1 candidates show minimal expression in matching normal tissue types and vital organs. To our knowledge, the majority (93%) of identified targets were novel. Previously identified targets included CD276 and GPC3, and CAR T cells targeting these antigens have been evaluated in early phase clinical studies with an encouraging safety profile^42–44^. However, we classified these targets as Tier 2 targets based on expression in vital organs. This highlights that gene expression not necessarily correlates with protein expression^45^. Likewise, antigen density is critical for efficient target cell recognition by CAR T cells^46^. Thus, Tier 2 targets should not be a priori excluded, but require additional studies to further assess the risk of on-target/off-cancer toxicity. The employed algorithm to identify targets might have detected membrane associated proteins that are not expressed on the cell surface, and additional validation studies have to be conducted for individual targets. Of note, we believe that these proteins should not be excluded a priori, since mislocalization of proteins have been described in cancer^47^.

In the present study, we used the normal tissue expression from GTEx as a control for identifying CSE targets in pediatric cancer. A potential caveat of this approach is that GTEx samples were from adult tissues, which may not completely match the normal expression in children. An ongoing public initiative, the developmental GTEx project aimed at stablishing a molecular and data analysis resource for gene expression in multiple relatively healthy reference neonatal, pediatric, and adolescent tissues, may ultimately provide a more accurate normal control for the childhood cancer cohort (https://www.genome.gov/Funded-Programs-Projects/Developmental-Genotype-Tissue-Expression). Currently, finding an appropriate match to the normal developmental stage of a pediatric cancer type remains extremely challenging as reactivation of fetal oncoprotein and immature developmental processes have thus far revealed critical therapeutic vulnerabilities for developing immunotherapy or small molecule-based interference for childhood cancer^48^. For example, antibodies against the fetal antigen GD2, which is expressed by neuroblastoma, are now routinely used in the treatment of high-risk neuroblastoma, and GD2-CAR T cells have also shown promising results in early phase clinical studies^49,50^. The vast majority of the CSE targets we identified were due to aberrant expression at the gene level as only 9 targets (COL6A3, FN1, POSTN, TNC, VCAN, NRCAM, FYN, PICALM and CLSTN1) were due to alternative splicing. This may be related to the use of exons defined by Gencode v31 gene models, which limits our ability to find AS targets in novel isoforms. Future studies that incorporate novel isoform discovery with CSE analysis or other newly published methods such as Isoform peptides from RNA splicing for Immunotherapy target Screening (IRIS)^51^ followed by validation using proteomics databases may further expand the repertoire of AS targets. Our studies demonstrated that FN1 and COL11A1, targets that are associated with the matrisome, are expressed by cancer cells. For adult cancers, these are also expressed by stromal and/or endothelial cells of the tumor microenvironment (TME)^52,53^, and additional studies are needed to investigate this for pediatric cancers.

Our analysis focuses on identifying CSEs as candidate immunotherapeutic targets themselves rather than on peptides derived from these exons that are presented by MHC molecules^54^. We decided on this approach so that the candidate targets can be broadly recognized by CAR T cells. In contrast, HLA-restricted peptides can, in general, only be targeted with MHC-restricted, αβ T-cell receptor (TCR) T cells^55^, although antibody based approaches are also being developed^56^. The CSE targets we identified are in genes with diverse biological functions. In some cases, gene-level overexpression in pediatric cancer is known without the knowledge of the expressed isoform. For example, TNC, a glycoprotein, is known to be highly expressed in pediatric EPN and HGGs^57,58^. Our exon-based analysis also identified several splice variants (e.g., C domain of TNC, EDB, COL6A3) known to be enriched in adult cancers^29,32,40^. This has broad therapeutic implications for pediatric cancers, since exon-targeted immunotherapies or imaging approaches that are currently being developed for adult cancer could be readily applied to pediatric cancer. Additional CSEs have been reported for FN1 and TNC.^28,30^ While we excluded the extradomain A of FN1 as an CSE due to expression in several normal tissues (**Supplementary Fig 6A**), we identified additional CSEs for TNC, which have been reported in adult cancer,^30^ including the CSE that encode the D domain of TNC (**Supplementary Fig 6B**).

We took advantage of PDX models of common pediatric solid tumors (OS, EWS, RMS) to quantify the expression of VCAN1, COL11A1, and EDB. Using orthogonal assays such as flow cytometry, IHC, and RT-qPCR, we were able to consistently detect the expression of these splice variants and gene expression, highlighting the robustness of our analytical approach. Although gene expression does not always correlate with protein expression, we found overall good correlation between our conducted assays.

We and other investigators had recently shown in preclinical models that EDB-CAR T-cells have potent antitumor activity, targeting not only tumor cells but also endothelial cells of the tumor vasculature^19,20^. These studies suggested that ECM proteins like FN1 that adhere to the cell surface can serve as CAR targets. To explore if this also applies to other ECM proteins, we generated CAR T cells specific for COL11A1, which was expressed at high levels in OS and CPC. COL11A1-CAR T cells recognized and killed COL11A1-positive tumor cells *in vitro* and had potent antitumor activity *in vivo* in two pediatric sarcoma xenograft models. While tumors eventually recurred, treated mice had a survival advantage. The survival advantage was particularly striking in our LM7 OS model, which expressed COL11A1 at high levels. We observed limited CAR T cell persistence and decreased expression of COL11A1 in recurring tumors, and future studies are required to gain additional insight into the mechanism of recurrence, and explore the therapeutic benefit of COL11A1-CAR T cells that are further genetically modified to enhance their effector function. Our finding that COL11A1 can serve as a CAR target should have broad implications since COL11A1 is also expressed in adult cancers with poor prognosis, such as pancreatic adenocarcinoma^59^, and has been proposed as a novel biomarker.^52^

In conclusion, by performing a comprehensive data mining using the rich RNA-seq data sets, we have demonstrated that the surfaceome/matrisome of pediatric solid and brain tumors contains cancer-specific exons that can serve as candidates for cancer immunotherapy. We identified and validated candidate targets with orthogonal assays and demonstrated that CAR T cells constructed from these targets have potent antitumor activity. Validation of consistent expression of target genes and to exclude epitope masking due to the tertiary structure of the protein in individual tumor cells is critical, which may involve performing IHC of primary patient samples and evaluating gene expression at single cell level by re-analyzing appropriate scRNA-seq data set as demonstrated in our validation of COL11A1. While we focused here on CAR T cells, the identified antigen could serve as targets of mAbs, immunocytokines or antibody drug conjugates. The full data set, explorable online (https://cseminer.stjude.org/), provides a comprehensive roadmap for developing future immunotherapies in childhood cancer.

## METHODS

### RNAseq data source

Solid and brain pediatric tumor RNA-seq data were downloaded from the St. Jude Cloud^9^ (https://platform.stjude.cloud/data/cohorts/pediatric-cancer) for St. Jude/Washington University Pediatric Cancer Genome Project (PCGP) and St. Jude’s Clinical Genomics (ClinGen) program. NCI TARGET data were downloaded from dbGaP under accession phs000218. RNA-seq data from the normal tissues were generated by the Genotype-Tissue Expression (GTEx) consortium^60^ and downloaded from the GTEx portal (https://gtexportal.org release v7).

### RNAseq mapping and exon quantification

RNA-seq reads were mapped using the STAR 2.7.1a program in two-pass mode^61^ to the human hg38 genome build using Gencode v31 primary assembly gene annotation gene models. Annotation of the exons status was based on APPRIS^62^. We used htseq^63^ to quantify the exon level expression and converts gene transfer format (GTF) to exon-specific GTF. Specifically, we ran htseq-count using the parameters below to ensure reads with multiple mapping were incorporated when measuring expression of exons that have high-fidelity paralogous duplications (see **Supplementary Fig 7** for an example):


htseq-count -f bam -r pos -a 0 -s no -m union -t exon –nonunique all


If a read spans a splice junction, it would be counted for both exons, which could potentially lead to overestimation of expression level of a short exon. Read counts were further normalized to FPKM (fragments per kilobase of transcript per million mapped reads) and to mitigate the potential bias on short exons, we used read length (instead of exon length) for normalizing exons that are shorter than the read length. The source code and documentation for each analysis can be found in GitHub (https://github.com/shawlab-moffitt/CSEminer-manuscript/tree/main/1_rnaseq_mapping_exonquant).

### Selection of cancer-specific exons (CSE) by performing tumor-vs-normal differential expression analysis

Differential expression was performed based on Wilcoxon rank-sum test. Let X1,…,Xn be the exon expression of tumor tissues, and Y1,…,Yn be the exon expression of normal tissue.


$$U=\sum_{i=1}^{n}\sum_{j=1}^{m}S\left(X_{i},Y_{j}\right)$$

With

$$S(X,Y)=\left\{\begin{array}{l} 1,\;\mathrm{if}\;Y<X\\ \frac{1}{2},\;\mathrm{if}\;Y=X\\ 0,\;\mathrm{if}\;Y>X \end{array}\right.$$

We then estimated the Z-score by a normal approximation of the U-statistics. Let n1 be the length _tissue._ of the number of samples from a cancer type, and let n2 be the number of samples from a normal $m_{U}$ *and* $\sigma_{U}$_are the mean and standard deviation of U._


$$m_{U}=\frac{n1n2}{2}$$

$$\sigma_{U}=\sqrt{\frac{n1n2(n1+n2+1)}{12}}$$

$$z=\frac{U-m_{U}}{\sigma_{U}}$$

To provide a meta-comparison of consistently differentially expressed exons, we applied Stouffer’s meta-analysis to combine k pairs of disease to normal comparison.

$$\mathrm{Composite}\;\mathrm{Zscore}∼\frac{\sum_{i=1}^{k}w_{i}Z_{i}}{\sqrt{\sum_{i}^{k}w^{2}}}$$

with the weight defined as the median percentile rank differential between tumor and normal tissue.


w=median(PercentileRank(X))-median(PercentileRank(Y))

Solid tumors and brain tumors were analyzed separately. A candidate CSE exon is required to have a composite Z-score >1 and above-median expression in at least one tumor type. To ensure low expression in normal tissues, candidates are also required to have ≤5 normal tissues expressed above the median level.

### Protein annotation for CSE targets

We retained targets encoding surfaceome or matrisome based on the following data sets: The Cell Surface Protein Atlas^64^, the MGI GO annotation^65^, the human protein atlas^66^, MatrixDB^67^, and the compartment database^68^. We started by using Ensembl for the reference gene annotation which includes 59,088 genes and 226,950 transcripts. Genes that are tumor suppressors or known to be DNA binding, such as transcription factors and chromatin regulators, were filtered out. This resulted in 67,472 exons from 2,273 genes encoding extracellular or surfaceome proteins. The transmembrane information was predicted based on TMHMM server 2.0^10^. Intersecting this reference surfaceome or matrisome gene set with CSE candidates resulted in 249 genes encoding 3,957 CSEs. Oncofetal annotation was derived from text mining from GeneCard followed by manual curation. Genes associated with tumor suppressors, transcription factor, epigenetic factors, kinases, cell differentiation factors, cytokine growth factors, and gene with the homeodomain were downloaded from MsigDB^69^ (**Supplementary Table 1**). The status of 82 tumor suppressors in pediatric cancer were verified using mutation data on PeCan portal (https://pecan.stjude.org) which were curated from >5,000 pediatric cancer patients.

### Curation of expression specificity and splicing pattern of CSE targets

CSEs were characterized as either gene-level or AS exon targets based on the following criteria: a) transcripts with <40% CSE coverage were subjected to further examination as candidates; and b) AS targets were required to match an alternatively spliced transcript in the reference database.

Candidate CSE targets for a cancer type profiled by multiple data resources (e.g. OS was profiled by ClinGen, PCGP and TARGET) required cross-validation of their expression in individual data resource to minimize the impact of coverage bias caused by different RNA-seq protocol. Additionally, verification of target expression in a proteomics database was required. In this study we used proteomics data generated from PDX models of pediatric solid tumors^15^ and brain tumors^14^ for this purpose. Additionally, any candidate targets identified in brain tumor which also exhibit high expression in normal brain (medium expression above 3^rd^ quartile) or a significant bias for exon position in GTEx data set (P value <0.01 for Pearson correlation between exon expression and exon number) are removed. The exon position bias check removes false positives caused by the 3’ bias in mRNA-seq protocol used by GTEx

Those targets that passed the QC check described above were further classified as Tier 1 or Tier 2 based on their expression status in normal tissues. Specifically, a Tier 1 candidate is expected to pass the following check: 1) absence of high expression in normal tissues paired to the tumor as follows: ACT/Adrenal, WLM/Kidney, RHB/Muscle, RB/Nerve, Mel/Skin; 2) low expression level in normal bone marrow samples for gene-level targets; and 3) low protein expression in normal tissues based on the GTEx proteomics data. Those that failed in any of these checks were classified as Tier 2. Details of analysis on normal bone marrow samples and GTEx Proteomics data are described below. Evaluation of expression level in normal bone marrow samples is needed because these normal samples were not profiled by GTEx. Apng the method described in reference 13^13^ we determined the expression level of the target genes using data from reference 67^70^. As microarray data were generated for gene-level expression, we were not able to determine the expression status in bone marrow for AS targets.

To ensure that low protein expression of Tier 1 targets in normal tissues, we analyzed the GTEx proteomics data from http://gbsc-share.stanford.edu/GTEx_raw_files. We first normalized the peptide spectral matches (nPSM) to the exon length of the peptide-protein-sequence coded within each exon and identified a bimodal distribution of the nPSMs and used the optim function to identify a cutoff point separating the two modes (**Supplementary Fig 8**). For each protein, we calculated an average nPSM based on the exon information and categorized candidates with high normal GTEx pro abundance which are subsequently downgraded to Tier 2.

### Tumor versus normal score for pan-cancer scatter plot

We generated an expression score for each exon to enable visual inspection of expression level in tumor versus normal for all candidate targets on the pan-target scatter plot. First, we calculated a binned score based on quartiles of exons that are above 1 FPKM. Exons below 1 FPKM were set to 0. We then calculated the mean of binned score for each tumor type and normal tissue type. Finally, we used the average of binned score across all tumor types and all normal tissue types to set the tumor score and normal score, respectively.

$$\mathrm{Binned}\;\mathrm{Score}(Xi)=\left\{\begin{array}{l} 0,\;\mathrm{if}\;\mathrm{quartile}(\mathrm{median}(Xi))∼\;”\mathrm{1st}\;\mathrm{quartile}”\;\\ 1\;\mathrm{if}\;\mathrm{quartile}(\mathrm{median}(Xi))∼”2\mathrm{nd}\;\mathrm{quartile}”\;\\ 2\;\mathrm{if}\;\mathrm{quartile}(\mathrm{median}(Xi))∼\;”\mathrm{3rd}\;\mathrm{quartile}”\;\\ 3,\;\mathrm{if}\;\mathrm{quartile}(\mathrm{median}(Xi))∼”4\mathrm{th}\;\mathrm{quartile}”\; \end{array}\right.$$

$Average\;Binned\;Score(X)=\frac{\sum_{i=1}^{n}BinnedScore(Xi)}{n},\;n$ represents the number of tumor types or normal tissue types

### Validation of AS targets using full-length transcriptome sequencing data of OS patient samples

We generated libraries and performed Iso-Seq sequencing for 3 OS patient samples on a PacBio RSII instrument. The raw data files were processed according to the PacBio Isoseq3 pipeline which utilizes a number of command line tools provided in PacBio SMRT Tools v10.2 (https://www.pacb.com/support/software-downloads/). The pipeline generates non-redundant full-length (FL) transcripts in the following steps for each tumor sample: (i) compute consensus sequences and read quality, (ii) remove primers and adapters, (iii) remove polyA tail and artificial concatemers, (iii) de novo isoform-level clustering, (iv) minimap2 aligns FL transcripts to human reference (GENCODEv40), (v) transcripts were collapsed based on genomic mapping abundance was estimated and GTF annotation file generated, (vi) sqanti3 performed transcript classification and generated a reference corrected transcriptome fasta file. The GTF file was searched for transcripts matching the gene target region coordinates.

### Splice variant analysis

To determine whether the splice variants affect the expression of alternatively spliced exons identified in the 9 genes, we obtained genomic variants for 504 tumor samples which have their WGS data available on St Jude Cloud Genomic Platform (https://platform.stjude.cloud/). To find splice variants, we queried the tumor variant files which contain both somatic and germline variants for those located within 10bp of splice acceptor and donor sites of the 9 AS exons in COL6A3 (chr2:237378636–237379235), FN1 (chr2:215392931–215393203), POSTN (chr13:37574572–37574652), TNC (chr9:115048260–115048532), VCAN(chr5:83519349–83522309), NRCAM (chr7:108191254–108191283), FYN (chr6:111699515–111699670), PICALM (chr11:85990250–85990378), and CLSTN1 (chr1:9756481–9756510). No variants were found for COL6A3, POSTN, TNC, NRCAM, FYN, PICALM, and CLSTN1. For the remaining two genes (FN1 and VCAN), no association between variants and expression level was detected based on 1-sided t-test, not surprising given the very low variant prevalence (1 out of 300 for FN1 and 21 out of 225 for VCAN) in tumors with median and high-level expression.

### Proteomics data analysis

To examine the protein-coding potential of candidate exons, we leveraged existing mass spectrometry profiling data sets generated from cancer cells relevant to our study. These included the deep mass spectrometry profiling of RMS^15^, brain tumors^14^, and patient derived xenograft (PDX) models were downloaded from the St. Jude proteomics facility and Clinical Proteomic Tumor Analysis Consortium (CPTAC). MS raw data were processed using the COMET software (http://comet-ms.sourceforge.net/)^71^, an open-source fast MS/MS sequence database search tool using a fast cross-correlation algorithm^72^. Briefly, raw MS files were searched against the human database downloaded from UniProt (52,490 entries) with Met oxidation as a dynamic modification. Search parameters were precursor and product ion mass tolerance (6 ppm and 10 ppm, respectively), fully tryptic restriction, two maximal missed cleavages, static TMT modification (+229.162932 Da on N-termini and Lys residues), dynamic Met oxidation (+15.99492 Da), three maximal dynamic modification sites, and the consideration of a, b, and y ions. Peptide-spectrum matches (PSMs) were filtered by seven amino acids minimal peptide length, mass accuracy (~3 ppm), and matching scores cutoff of < 2 xcorr and Δxcorr > 0.1. The visualization of the mass-spectrometry peaks was performed on the Msviewer^73^.

### Tumor cell lines

143B (OS), CRL-2061 and CCL-136 (RMS), and A673 (EWS) cell lines were purchased from the American Type Tissue Collection (ATCC). The lung metastatic osteosarcoma cell line LM7 was kindly provided by Dr. Eugenie Kleinerman (MD Anderson Cancer Center, Houston, TX) in 2011. Primary fibroblast (Fib) cell lines from healthy donors were previously established^74^. The generation of all tumor cell lines expressing an enhanced green fluorescence protein firefly luciferase fusion gene (GFP.ffluc) was previously described^18^. The COL11A1 KO 143B cell line was generated by St. Jude’s Center for Advanced Genome Engineering (CAGE) using CRISPR/Cas9 gene-editing technology. All cell lines were grown in DMEM or RPMI (Fisher Sci SH30022.01; Genclone 25–506N) supplemented with 10% fetal bovine serum (FBS; GE Healthcare Life Sciences HyClone, SH3008803) and 2 mM Glutamax (Invitrogen, 35050061). Cell lines were authenticated using ATCC’s human STR profiling cell authentication service. Cell lines were free of mycoplasma contamination, and routinely checked for Mycoplasma by the MycoAlert Mycoplasma Detection Kit (Lonza, LT07–118).

### Patient-derived xenograft samples

Orthotopic patient-derived xenograft samples, collected under the Molecular Analysis of Solid Tumors (MAST) protocol, were provided by the Childhood Solid Tumor Network (CSTN) collection at St. Jude (https://cstn.stjude.cloud/search/)^75^. The gene expression from the primary patient tumors and PDX models are highly correlated (R>0.8) except for patient samples with low tumor purity (purity <20%) due to the high-level admixture of gene expression in stromal cells (see Extended Data Figure 3 of the CSTN manuscript)^75^. All samples were handled in accordance with CSTN policy including DNA profiling for short tandem repeat validation to confirm orthotopic (O)-PDX models between passages. Samples were hand homogenized in PBS (Lonza, 17–512F) with 1% FBS (HyClone, SH3008803) and filtered twice through polystyrene test tubes with cell strainer caps (Falcon, 352235). Single cell suspension was used for both flow cytometry and real-time PCR.

### Primary sarcoma tissue sections

After St. Jude Institutional Review Board approval, deidentified archival formalin-fixed paraffin-embedded tissue blocks from clinical patient tumor samples were cut and H&E-stained sections were reviewed for correct diagnosis and tumor content by a pediatric pathologist (SCK). Matched unstained tumor sections were then stained. Samples were delineated into 3 categories based on expression levels: high, low, and negative based on normal tissue controls.

### Immunohistochemistry

To detect COL11A1 expression, IHC was performed on Ventana Discovery Ultra autostainer (Roche, Indianapolis, IN) with the following protocol and reagents. Vial of mAb anticol11A1 (Oncomatryx, High Concentration 2.3 mg/mL Rabbit monoclonal (Clone 1e8.33)). All reagents were provided by Roche, Indianapolis IN: Samples underwent heat-induced epitope retrieval, (Cell Conditioning Solution ULTRA CC1 (950–224, Roche)) for 32 minutes; the primary antibody was incubated for 30 minutes per manufacturers instruction; followed by DISCOVERY OmniMap anti-Rt HRP (760–4457; Roche), DISCOVERY ChromoMap DAB kit (760–159; Roche), Hematoxylin II (790–2208; Roche), and Bluing reagent (790–2037; Roche) were used for visualization. All samples (PDX, xenograft, primary, normal) were stained in along with positive control (LM7 xenograft) and negative control xenograft (143B COL11A1 KO xenograft), grown subcutaneously in NSG female mice, and tumors were harvested when they reached a size of 1000 mm^3^. Isotype controls were used as well.

### Reverse transcription quantitative PCR

mRNA extraction from single cell suspensions of cultured cell lines (<1×10^7^ cells) and PDX samples was performed using the Maxwell RSC simplyRNA Blood kit (Promega AS1380) on a Maxwell RSC machine. RT-qPCR was performed according to the manufacturer’s instructions with 10 ng of RNA and 200 nM of primers using the Power SYBR Green RNA-to-C_T_ 1-Step Kit (Thermo Fisher Scientific, 4389986) on an Applied Bioscience QuantStudio 6 Flex machine, and analyzed using QuantStudio software (Thermo Fisher Scientific). GAPDH primers were purchased from IDT (PrimeTime qPCR Primers, human GAPDH, Hs.PT.39a.22214836). Primers (IDT) were designed to detect EDB and COL11A1 using the NCBI Primer-BLAST tool.

EDB domain of FN1 Forward: 5’-CCC CAA CTC ACT GAC CTA AGC-3’

EDB domain of FN1 Reverse: 5’-CTG CCG CAA CTA CTG TGA TG-3’

COL11A1 Forward: 5’- CAG ACG GAG GCA AAC ATC GT-3’

COL11A1 Reverse: 5’-TCA TTT GTC CCA GAA ACA TGC C-3’

### Generation of retroviral vectors

In-fusion cloning (Takara Bio, 638947) was used to generate the COL11A1-CAR with a CD28 costimulatory domain and IgG1 short hinge using our retroviral vector as a template, which encodes a EphA2-CAR.CD28ζ expression cassette, a 2A sequence, and truncated CD19^76^. The COL11A1-specific scFv was derived from mAb 1e8.33^40^ and synthesized by GeneArt (Thermo Fisher Scientific). The non-functional COL11A1-CAR with mutated (mut) ITAMs was generated by using our retroviral vector encoding a CD28z.mut.CAR as a template^19^. The sequences of the final constructs were verified by sequencing (Hartwell Center, St. Jude Children’s Research Hospital). The generation of the EDB-CAR was described previously^19^. RD114-pseudotyped retroviral particles were generated by transient transfection of 293T cells as previously described^76^.

### Generation of CAR T cells

Human peripheral blood mononuclear cells (PBMCs) were isolated using Lymphoprep (Abbott Laboratories) from de-identified elutriation chambers of leukapheresis products obtained from St. Jude’s donor center or obtained from healthy donors under an IRB approved protocol at St. Jude Children’s Research Hospital, after informed consent was obtained in accordance with the Declaration of Helsinki. To generate CAR T cells, we used our previously described standard protocol^76^. Briefly, PBMCs were stimulated on treated non-tissue culture 24-well plates, which were precoated with CD3 and CD28 antibodies (Miltenyi, #130-093-38, #130-093-375). Recombinant human IL-7 and IL-15 (IL-7: 10 ng/mL; IL-15: 5 ng/mL; PeproTech P13232, 40933) were added to cultures the next day. On day 2, CD3/CD28-stimulated T cells (2.5×10^5^ cells/well) were transduced with RD114-pseudotyped retroviral particles on RetroNectin (Takara)-coated plates in the presence of IL-7 and IL-15. On day 5, transduced T cells were transferred into new tissue culture 24-well plates and subsequently expanded with IL-7 and IL-15. Non-transduced (NT) T cells were prepared in the same way except for no retrovirus was added. All experiments were performed 7–14 days post-transduction using unsorted ‘bulk’ CAR T cells. Biological replicates were performed using PBMCs from different healthy donors.

### Flow cytometry

A FACSCanto II (BD) instrument was used to acquire flow cytometry data, which was analyzed using FlowJo v10 (FlowJo). For surface staining of CAR T cells, samples were washed with and stained in PBS (Lonza) with 1% FBS (HyClone). For all experiments, matched isotypes or known negatives (e.g., NT T-cells, KO cell lines, known antigen-negative cell lines) served as gating controls along with positive control (e.g., anti-CD4 in all colors). LIVE/DEAD^®^ Fixable Aqua Dead Cell Stain Kit (Invitrogen) or DAPI was used as a viability dye. T-cells were evaluated for CAR expression at multiple time points post-transduction with an anti-human IgG, F(ab’)2 fragment specific-AF647; anti-mouse IgG, F(ab’)2 fragment specific AF647, (Jackson ImmunoResearch 109-605-006, 115-605-006). Transduction was also confirmed with anti-CD19-PE (clone J3–119, Beckman Coulter, IM1285U, 0.5μL/100μL).

For detecting EDB expression, we used a recombinant L19 mAb as previously described^19,39^, which synthesized by ThermoFisher based on publicly available sequences, which are published^19^. Anti-COL11A1 (Invitrogen, PA5-101300) and anti-VCAN (Novus NBP2-22408) were used to detect the respective antigens. Antibodies were conjugated using Lightning-Link^®^ Labeling Kits (Novus Bio) according to the manufacturer’s instructions. Cells were prepared for surface staining at 1:300 antibody dilution based on manufactures instructions (COL11A1). All cell lines were analyzed at same voltages for each antibody in 3 technical replicates for accurate comparison. Mean of the analyses was determined and graphed accordingly.

### Co-culture assay

1×10^6^ CAR T-cells were co-cultured with 5×10^5^ LM7, A673, 143B, CRL-2061, or CCL-136 tumor cells, or 3×10^5^ primary fibroblasts without the provision of exogenous cytokine. CAR T-cells cultured without tumor cells served as controls. After 48 hours, media was collected and frozen for later analysis. Cytokines were measured using IFNγ ELISA kits (R&D Systems, DIF50C) according to the manufacturer’s instructions.

### Cytotoxicity assay

In a tissue culture-treated 96-well plate, GFP.ffluc-expressing tumor cells (12,500 A673, 143B, KO 143B, CRL2061, CCL-136, or 15,000 LM7) or 15,000 fibroblasts were co-cultured with serial dilutions of NT or CAR T cells. Each condition was plated in triplicate. After 3 days, 0.6 mg of D-luciferin (Perkin Elmer, 122799–10) was added to each well and luminescence was evaluated using an Infinite^®^ 200 Pro MPlex plate reader (Tecan) to assess the number of viable cells in each well. Percent live tumor cells were determined by the following formula: (sample-media alone)/(tumor alone-media alone)*100.

### Xenograft mouse models

Animal experiments followed a protocol approved by the St. Jude Institutional Animal Care and Use Committee. All experiments utilized 6–8-week NOD-scid IL2Rgammanull (NSG) mice obtained from St. Jude’s NSG colony.

#### Intraperitoneal tumor models:

Mice were injected intraperitoneally (i.p.) with 1×10^6^ LM7.GFP.ffLuc tumor cells, and on day 7 received a single i.v. dose of 3×10^6^ T cells. For survival experiment, mice were euthanized when they reached i) the bioluminescence Flux endpoint of 2×10^10^ on two consecutive measurements, and ii) they met physical euthanasia criteria (significant weight loss, signs of distress). To test for antigen loss variants, mice were injected with 1×10^6^ LM7 tumor cells, and on day 7 received a single i.v. dose of 3×10^6^ GFP.ffLuc-expressing T cells. Mice were euthanized at day 65 and tumors were harvested in the peritoneum for COL11A1 IHC.

#### Subcutaneous tumor models:

Mice were injected subcutaneously (s.c.) with 1×10^6^ A673 tumor cells in Matrigel (Corning; 1:1 diluted in PBS). On day 7, mice received a single i.v. dose of 1×10^6^ T cells via tail vein injection. Tumor growth was assessed by serial caliper measurements from a third-party animal technician to allow for a blinded study. Mice were euthanized when i) they met physical euthanasia criteria (significant weight loss, signs of distress), ii) the tumor burden was approximately 3,000 mm^3^, or iii) recommended by St. Jude veterinary staff.

### Bioluminescence imaging

Mice were imaged as described previously.^19^ Briefly, they were injected i.p. with 150 mg/kg of D-luciferin 5–10 minutes before imaging, anesthetized an induction chamber (2–3% isoflurane, with oxygen), after which placed in the imaging instrument and fitted with a nose cone connected to a vaporizer to maintain isoflurane (1.0–2.5%) during the procedure. Images were acquired on a Xenogen IVIS-200 imaging system. The photons emitted from the luciferase-expressing tumor cells were quantified using Living Image software (Caliper Life Sciences).

### Statistical analysis

All experiments were performed at least in triplicates. For comparison between two groups, two-tailed t-test was used. For comparisons of three or more groups, values were log transformed as needed and analyzed by ANOVA with Tukey’s post-test. Survival was analyzed by Kaplan-Meier method and by the log-rank test. Statistical analyses were conducted with Prism software (Version 9.0.0, GraphPad Software).

### Reagent and protocol availability

Contact Jinghui Zhang at jinghui.zhang@stjude.org or Stephen Gottschalk at stephen.gottschalk@stjude.org.

## Extended Data

## Data Availability

The raw RNA-seq data for PCGP and St Jude ClinGen samples are available on St Jude Cloud Genomics Platform (https://platform.stjude.cloud/data/cohorts/pediatric-cancer) under the accessions SJC-DS-1001, SJC-DS-1003, SJC-DS-1004 and SJC-DS-1007. NCI TARGET data are available in dbGaP under accession phs000218. Patient sample IDs and their associated accessions can be found at Supplementary Table 4.

## References

[1] Wedekind M.F., Denton N.L., Chen C.Y. & Cripe T.P. Pediatric Cancer Immunotherapy: Opportunities and Challenges. Paediatr Drugs 20, 395–408 (2018). 29948928 10.1007/s40272-018-0297-x PMC6153971

[2] Wagner J., Wickman E., DeRenzo C. & Gottschalk S. CAR T Cell Therapy for Solid Tumors: Bright Future or Dark Reality? Molecular therapy : the journal of the American Society of Gene Therapy 28, 2320–2339 (2020). 32979309 10.1016/j.ymthe.2020.09.015 PMC7647674

[3] Rafiq S., Hackett C.S. & Brentjens R.J. Engineering strategies to overcome the current roadblocks in CAR T cell therapy. Nat Rev Clin Oncol 17, 147–167 (2020). 31848460 10.1038/s41571-019-0297-y PMC7223338

[4] Brohl A.S., Immuno-transcriptomic profiling of extracranial pediatric solid malignancies. Cell reports 37, 110047 (2021). 34818552 10.1016/j.celrep.2021.110047 PMC8642810

[5] Bosse K.R., Identification of GPC2 as an Oncoprotein and Candidate Immunotherapeutic Target in High-Risk Neuroblastoma. Cancer Cell 32, 295–309 e212 (2017). 28898695 10.1016/j.ccell.2017.08.003 PMC5600520

[6] Heitzeneder S., Pregnancy-Associated Plasma Protein-A (PAPP-A) in Ewing Sarcoma: Role in Tumor Growth and Immune Evasion. J Natl Cancer Inst 111, 970–982 (2019). 30698726 10.1093/jnci/djy209 PMC6748813

[7] Perna F., Integrating Proteomics and Transcriptomics for Systematic Combinatorial Chimeric Antigen Receptor Therapy of AML. Cancer Cell 32, 506–519 e505 (2017). 29017060 10.1016/j.ccell.2017.09.004 PMC7025434

[8] Majzner R.G., CAR T Cells Targeting B7-H3, a Pan-Cancer Antigen, Demonstrate Potent Preclinical Activity Against Pediatric Solid Tumors and Brain Tumors. Clinical cancer research : an official journal of the American Association for Cancer Research 25, 2560–2574 (2019). 30655315 10.1158/1078-0432.CCR-18-0432 PMC8456711

[9] McLeod C., St. Jude Cloud: A Pediatric Cancer Genomic Data-Sharing Ecosystem. Cancer discovery 11, 1082–1099 (2021). 33408242 10.1158/2159-8290.CD-20-1230 PMC8102307

[10] Krogh A., Larsson B., von Heijne G. & Sonnhammer E.L. Predicting transmembrane protein topology with a hidden Markov model: application to complete genomes. J Mol Biol 305, 567–580 (2001). 11152613 10.1006/jmbi.2000.4315

[11] Thul P.J. & Lindskog C. The human protein atlas: A spatial map of the human proteome. Protein Sci 27, 233–244 (2018). 28940711 10.1002/pro.3307 PMC5734309

[12] Chautard E., Fatoux-Ardore M., Ballut L., Thierry-Mieg N. & Ricard-Blum S. MatrixDB, the extracellular matrix interaction database. Nucleic Acids Res 39, D235–240 (2011). 20852260 10.1093/nar/gkq830 PMC3013758

[13] Schreiner P., Velasquez M.P., Gottschalk S., Zhang J. & Fan Y. Unifying heterogeneous expression data to predict targets for CAR-T cell therapy. Oncoimmunology 10, 2000109 (2021). 34858726 10.1080/2162402X.2021.2000109 PMC8632331

[14] Petralia F., Integrated Proteogenomic Characterization across Major Histological Types of Pediatric Brain Cancer. Cell 183, 1962–1985 e1931 (2020). 33242424 10.1016/j.cell.2020.10.044 PMC8143193

[15] Stewart E., Identification of Therapeutic Targets in Rhabdomyosarcoma through Integrated Genomic, Epigenomic, and Proteomic Analyses. Cancer Cell 34, 411–426 e419 (2018). 30146332 10.1016/j.ccell.2018.07.012 PMC6158019

[16] Shrestha B., Human CD83-targeted chimeric antigen receptor T cells prevent and treat graft-versus-host disease. The Journal of clinical investigation 130, 4652–4662 (2020). 32437331 10.1172/JCI135754 PMC7456225

[17] Nguyen P., Route of 41BB/41BBL Costimulation Determines Effector Function of B7-H3-CAR.CD28ζ T Cells. Mol Ther Oncolytics 18, 202–214 (2020). 32728609 10.1016/j.omto.2020.06.018 PMC7369352

[18] Kakarla S., Antitumor effects of chimeric receptor engineered human T cells directed to tumor stroma. Molecular therapy : the journal of the American Society of Gene Therapy 21, 1611–1620 (2013). 23732988 10.1038/mt.2013.110 PMC3734659

[19] Wagner J., Antitumor Effects of CAR T Cells Redirected to the EDB Splice Variant of Fibronectin. Cancer immunology research 9, 279–290 (2021). 33355188 10.1158/2326-6066.CIR-20-0280 PMC7925432

[20] Xie Y.J., Nanobody-based CAR T cells that target the tumor microenvironment inhibit the growth of solid tumors in immunocompetent mice. Proceedings of the National Academy of Sciences of the United States of America 116, 7624–7631 (2019). 30936321 10.1073/pnas.1817147116 PMC6475367

[21] Li W., Redirecting T Cells to Glypican-3 with 4–1BB Zeta Chimeric Antigen Receptors Results in Th1 Polarization and Potent Antitumor Activity. Human gene therapy 28, 437–448 (2017). 27530312 10.1089/hum.2016.025 PMC5444493

[22] Trad R., Chimeric antigen receptor T-cells targeting IL-1RAP: a promising new cellular immunotherapy to treat acute myeloid leukemia. J Immunother Cancer 10(2022). 10.1136/jitc-2021-004222 PMC9272123 35803613

[23] Mori J.I., Anti-tumor efficacy of human anti-c-met CAR-T cells against papillary renal cell carcinoma in an orthotopic model. Cancer science 112, 1417–1428 (2021). 33539630 10.1111/cas.14835 PMC8019206

[24] Chinnasamy D., Local delivery of interleukin-12 using T cells targeting VEGF receptor-2 eradicates multiple vascularized tumors in mice. Clin.Cancer Res. 18, 1672–1683 (2012). 22291136 10.1158/1078-0432.CCR-11-3050 PMC6390958

[25] Arai Y., Myeloid Conditioning with c-kit-Targeted CAR-T Cells Enables Donor Stem Cell Engraftment. Molecular therapy : the journal of the American Society of Gene Therapy 26, 1181–1197 (2018). 29622475 10.1016/j.ymthe.2018.03.003 PMC5993968

[26] Vora P., The Rational Development of CD133-Targeting Immunotherapies for Glioblastoma. Cell Stem Cell 26, 832–844 e836 (2020). 32464096 10.1016/j.stem.2020.04.008

[27] Li N., CAR T cells targeting tumor-associated exons of glypican 2 regress neuroblastoma in mice. Cell Rep Med 2, 100297 (2021). 34195677 10.1016/j.xcrm.2021.100297 PMC8233664

[28] Rick J.W., Fibronectin in malignancy: Cancer-specific alterations, protumoral effects, and therapeutic implications. Semin Oncol 46, 284–290 (2019). 31488338 10.1053/j.seminoncol.2019.08.002 PMC6801036

[29] Johannsen M., The tumour-targeting human L19-IL2 immunocytokine: preclinical safety studies, phase I clinical trial in patients with solid tumours and expansion into patients with advanced renal cell carcinoma. Eur J Cancer 46, 2926–2935 (2010). 20797845 10.1016/j.ejca.2010.07.033

[30] Yilmaz A., Advances on the roles of tenascin-C in cancer. J Cell Sci 135(2022). 10.1242/jcs.260244 PMC9584351 36102918

[31] Jones P.L. & Jones F.S. Tenascin-C in development and disease: gene regulation and cell function. Matrix Biol 19, 581–596 (2000). 11102748 10.1016/s0945-053x(00)00106-2

[32] Silacci M., Human monoclonal antibodies to domain C of tenascin-C selectively target solid tumors in vivo. Protein Eng Des Sel 19, 471–478 (2006). 16928692 10.1093/protein/gzl033

[33] Kloeckener-Gruissem B., Novel VCAN mutations and evidence for unbalanced alternative splicing in the pathogenesis of Wagner syndrome. Eur J Hum Genet 21, 352–356 (2013). 22739342 10.1038/ejhg.2012.137 PMC3573191

[34] Annunen S., Splicing mutations of 54-bp exons in the COL11A1 gene cause Marshall syndrome, but other mutations cause overlapping Marshall/Stickler phenotypes. Am J Hum Genet 65, 974–983 (1999). 10486316 10.1086/302585 PMC1288268

[35] Sakazume S., GPC3 mutations in seven patients with Simpson-Golabi-Behmel syndrome. Am J Med Genet A 143A, 1703–1707 (2007). 17603795 10.1002/ajmg.a.31822

[36] Chapoval A.I., B7-H3: a costimulatory molecule for T cell activation and IFN-gamma production. Nat.Immunol. 2, 269–274 (2001). 11224528 10.1038/85339

[37] Zhao B., Immune checkpoint of B7-H3 in cancer: from immunology to clinical immunotherapy. J Hematol Oncol 15, 153 (2022). 36284349 10.1186/s13045-022-01364-7 PMC9597993

[38] Zhou Y., Single-cell RNA landscape of intratumoral heterogeneity and immunosuppressive microenvironment in advanced osteosarcoma. Nat Commun 11, 6322 (2020). 33303760 10.1038/s41467-020-20059-6 PMC7730477

[39] Pini A., Design and use of a phage display library. Human antibodies with subnanomolar affinity against a marker of angiogenesis eluted from a two-dimensional gel. J Biol Chem 273, 21769–21776 (1998). 9705314 10.1074/jbc.273.34.21769

[40] Garcia-Ocana M., Characterization of a novel mouse monoclonal antibody, clone 1E8.33, highly specific for human procollagen 11A1, a tumor-associated stromal component. International journal of oncology 40, 1447–1454 (2012). 22322826 10.3892/ijo.2012.1360

[41] Lange S., A Chimeric GM-CSF/IL18 Receptor to Sustain CAR T-cell Function. Cancer discovery 11, 1661–1671 (2021). 33563660 10.1158/2159-8290.CD-20-0896 PMC8292158

[42] Shi D., Chimeric Antigen Receptor-Glypican-3 T-Cell Therapy for Advanced Hepatocellular Carcinoma: Results of Phase I Trials. Clinical cancer research : an official journal of the American Association for Cancer Research 26, 3979–3989 (2020). 32371538 10.1158/1078-0432.CCR-19-3259

[43] Tang X., Administration of B7-H3 targeted chimeric antigen receptor-T cells induce regression of glioblastoma. Signal Transduct Target Ther 6, 125 (2021). 33767145 10.1038/s41392-021-00505-7 PMC7994554

[44] Vitanza N.A., Intraventricular B7-H3 CAR T cells for diffuse intrinsic pontine glioma: preliminary first-in-human bioactivity and safety. Cancer discovery (2022). 10.1158/2159-8290.CD-22-0750 PMC9827115 36259971

[45] Vogel C. & Marcotte E.M. Insights into the regulation of protein abundance from proteomic and transcriptomic analyses. Nat Rev Genet 13, 227–232 (2012). 22411467 10.1038/nrg3185 PMC3654667

[46] Majzner R.G., Tuning the Antigen Density Requirement for CAR T-cell Activity. Cancer discovery 10, 702–723 (2020). 32193224 10.1158/2159-8290.CD-19-0945 PMC7939454

[47] Wang X. & Li S. Protein mislocalization: mechanisms, functions and clinical applications in cancer. Biochimica et biophysica acta 1846, 13–25 (2014). 24709009 10.1016/j.bbcan.2014.03.006 PMC4141035

[48] Behjati S., Gilbertson R.J. & Pfister S.M. Maturation Block in Childhood Cancer. Cancer discovery 11, 542–544 (2021). 33589423 10.1158/2159-8290.CD-20-0926

[49] Yu A.L., Anti-GD2 antibody with GM-CSF, interleukin-2, and isotretinoin for neuroblastoma. N.Engl.J.Med. 363, 1324–1334 (2010). 20879881 10.1056/NEJMoa0911123 PMC3086629

[50] Del Bufalo F., GD2-CART01 for Relapsed or Refractory High-Risk Neuroblastoma. The New England journal of medicine 388, 1284–1295 (2023). 37018492 10.1056/NEJMoa2210859

[51] Pan Y., IRIS: Discovery of cancer immunotherapy targets arising from pre-mRNA alternative splicing. Proceedings of the National Academy of Sciences of the United States of America 120, e2221116120 (2023). 37192158 10.1073/pnas.2221116120 PMC10214192

[52] Nallanthighal S., Heiserman J.P. & Cheon D.J. Collagen Type XI Alpha 1 (COL11A1): A Novel Biomarker and a Key Player in Cancer. Cancers (Basel) 13(2021). 10.3390/cancers13050935 PMC7956367 33668097

[53] Villa A., A high-affinity human monoclonal antibody specific to the alternatively spliced EDA domain of fibronectin efficiently targets tumor neo-vasculature in vivo. Int.J Cancer 122, 2405–2413 (2008). 18271006 10.1002/ijc.23408

[54] Kim G.B., Quantitative immunopeptidomics reveals a tumor stroma-specific target for T cell therapy. Science translational medicine 14, eabo6135 (2022). 36044599 10.1126/scitranslmed.abo6135 PMC10130759

[55] Kahles A., Comprehensive Analysis of Alternative Splicing Across Tumors from 8,705 Patients. Cancer Cell 34, 211–224 e216 (2018). 30078747 10.1016/j.ccell.2018.07.001 PMC9844097

[56] Yarmarkovich M., Cross-HLA targeting of intracellular oncoproteins with peptide-centric CARs. Nature 599, 477–484 (2021). 34732890 10.1038/s41586-021-04061-6 PMC8599005

[57] Korshunov A., Golanov A. & Timirgaz V. Immunohistochemical markers for prognosis of ependymal neoplasms. Journal of neuro-oncology 58, 255–270 (2002). 12187959 10.1023/a:1016222202230

[58] Qi J., Tenascin-C expression contributes to pediatric brainstem glioma tumor phenotype and represents a novel biomarker of disease. Acta Neuropathol Commun 7, 75 (2019). 31092287 10.1186/s40478-019-0727-1 PMC6518697

[59] Garcia-Pravia C., Overexpression of COL11A1 by cancer-associated fibroblasts: clinical relevance of a stromal marker in pancreatic cancer. PloS one 8, e78327 (2013). 24194920 10.1371/journal.pone.0078327 PMC3808536

[60] Consortium G.T. Human genomics. The Genotype-Tissue Expression (GTEx) pilot analysis: multitissue gene regulation in humans. Science 348, 648–660 (2015). 25954001 10.1126/science.1262110 PMC4547484

[61] Dobin A., STAR: ultrafast universal RNA-seq aligner. Bioinformatics 29, 15–21 (2013). 23104886 10.1093/bioinformatics/bts635 PMC3530905

[62] Rodriguez J.M., APPRIS: annotation of principal and alternative splice isoforms. Nucleic Acids Res 41, D110–117 (2013). 23161672 10.1093/nar/gks1058 PMC3531113

[63] Putri G.H., Anders S., Pyl P.T., Pimanda J.E. & Zanini F. Analysing high-throughput sequencing data in Python with HTSeq 2.0. Bioinformatics (2022). 10.1093/bioinformatics/btac166 PMC9113351 35561197

[64] Bausch-Fluck D., The in silico human surfaceome. Proceedings of the National Academy of Sciences of the United States of America 115, E10988–E10997 (2018). 30373828 10.1073/pnas.1808790115 PMC6243280

[65] Gene Ontology C. Gene Ontology Consortium: going forward. Nucleic Acids Res 43, D1049–1056 (2015). 25428369 10.1093/nar/gku1179 PMC4383973

[66] Uhlen M., A human protein atlas for normal and cancer tissues based on antibody proteomics. Mol Cell Proteomics 4, 1920–1932 (2005). 16127175 10.1074/mcp.M500279-MCP200

[67] Clerc O., MatrixDB: integration of new data with a focus on glycosaminoglycan interactions. Nucleic Acids Res 47, D376–D381 (2019). 30371822 10.1093/nar/gky1035 PMC6324007

[68] Binder J.X., COMPARTMENTS: unification and visualization of protein subcellular localization evidence. Database (Oxford) 2014, bau012 (2014). 24573882 10.1093/database/bau012 PMC3935310

[69] Liberzon A., The Molecular Signatures Database (MSigDB) hallmark gene set collection. Cell Syst 1, 417–425 (2015). 26771021 10.1016/j.cels.2015.12.004 PMC4707969

[70] Haferlach T., Clinical utility of microarray-based gene expression profiling in the diagnosis and subclassification of leukemia: report from the International Microarray Innovations in Leukemia Study Group. Journal of clinical oncology : official journal of the American Society of Clinical Oncology 28, 2529–2537 (2010). 20406941 10.1200/JCO.2009.23.4732 PMC5569671

[71] Eng J.K., Jahan T.A. & Hoopmann M.R. Comet: an open-source MS/MS sequence database search tool. Proteomics 13, 22–24 (2013). 23148064 10.1002/pmic.201200439

[72] Eng J.K., Fischer B., Grossmann J. & Maccoss M.J. A fast SEQUEST cross correlation algorithm. J Proteome Res 7, 4598–4602 (2008). 18774840 10.1021/pr800420s

[73] Baker P.R. & Chalkley R.J. MS-viewer: a web-based spectral viewer for proteomics results. Mol Cell Proteomics 13, 1392–1396 (2014). 24591702 10.1074/mcp.O113.037200 PMC4014294

[74] Gottschalk S., Generating CTL against the subdominant Epstein-Barr virus LMP1 antigen for the adoptive Immunotherapy of EBV-associated malignancies. Blood 101, 1905–1912 (2003). 12411306 10.1182/blood-2002-05-1514

[75] Stewart E., Orthotopic patient-derived xenografts of paediatric solid tumours. Nature 549, 96–100 (2017). 28854174 10.1038/nature23647 PMC5659286

[76] Yi Z., Prinzing B.L., Cao F., Gottschalk S. & Krenciute G. Optimizing EphA2-CAR T Cells for the Adoptive Immunotherapy of Glioma. Mol Ther Methods Clin Dev 9, 70–80 (2018). 29552579 10.1016/j.omtm.2018.01.009 PMC5852415

